# 26th Annual GP2A Medicinal Chemistry Conference & 32nd Journées Franco-Belges de Pharmacochimie

**DOI:** 10.3390/ph12020073

**Published:** 2019-05-16

**Authors:** Patrick Dallemagne, Christophe Rochais, Pascal Marchand, Thierry Besson

**Affiliations:** 1 Centre d’Etudes et de Recherche sur le Médicament de Normandie, Normandie Univ, UNICAEN, CERMN, F-14000 Caen, France; patrick.dallemagne@unicaen.fr (P.D.); christophe.rochais@unicaen.fr (C.R.); 2 Université de Nantes, Cibles et Médicaments des Infections et du Cancer, IICiMed, EA 1155, F-44000 Nantes, France; 3 Normandie Univ, UNIROUEN, INSA Rouen, CNRS, COBRA UMR 6014, F-76000 Rouen, France; thierry.besson@univ-rouen.fr

**Keywords:** medicinal chemistry, drug design, chemical tools

## Abstract

As a joint meeting, the 26th Medicinal Chemistry Conference of GP2A and 32nd Journées Franco-Belges de Pharmacochimie took place between 13th and 15th June at Asnelles sur Mer (Normandie, France), providing a unique opportunity for a wide group of European medicinal chemists to engage. Topics included chemical tools for medicinal chemistry, protein-protein interactions, epigenetics, natural product-inspired molecules, computer-aided drug design, and new strategies for the design and development of drugs. Abstracts of invited lectures, proffered young researcher communications, flash communications and posters presented during the meeting are collected in this report.

## 1. Aim and Scope of the Meeting

The Groupement des Pharmacochimistes de l’Arc Atlantique (GP2A) is a group of academic medicinal chemists working in universities and research institutes near the western coast of Europe (the “Atlantic Arc”). It was founded in 1992 with the aim of bringing together researchers in this area to exchange ideas and experiences. Historically, it has included members from France, Spain, Portugal, Ireland and the United Kingdom. It has recently expanded both geographically and in terms of research fields, now including researchers from areas that range from physical and pharmaceutical chemistry to molecular pharmacology.

The “Journées Franco-Belges de Pharmacochimie” (JFB) is a widely recognized annual medicinal chemistry meeting. This two-day symposium, whose first edition was held in 1986, aims to promote exchanges between medicinal chemists, mainly from France and Belgium. It is renowned for the advanced science presented, conviviality, and outstanding opportunities for senior and young scientists to exchange knowledge.

The aim of the annual conference is the exchange of ideas and experience, particularly amongst young researchers. This is achieved in two ways, through focused conferences and the facilitation of short exchange visits. Each year, we hold a meeting designed to bring together not only laboratory heads (Principal Investigators) but also postdocs and postgraduate research students (PhD students). At these meetings, everyone has the opportunity to present and discuss their latest research through invited lectures, oral communications and posters. In 2018, the joint conference was held in Asnelles sur Mer (Normandy, France) and was organized by members from the Universities of Caen and Rouen. The major research topics that were presented included chemical tools for medicinal chemistry, protein-protein interactions, epigenetics, natural product-inspired molecules, computer-aided drug design, and new strategies for the design and development of drugs. These presentations were in the forms of lectures by invited experts, proffered young researcher communications, posters with flash oral presentations and a large number of posters from young scientists, PhD students and postdoctoral researchers.

## 2. Inaugural Lesson

### Rational Development of a PET Radiopharmaceutical Probe

BarréLouisaCEA, University of Caen, 14032 Cannes, France; barre@cyceron.fr

Molecular imaging with PET is a rapidly emerging approach in oncology. This approach offers the potential to noninvasively determine tumor staging, make tumor response measurements, and characterize relevant drug targets for treatment. The aims of this presentation are to summarize the steps from preclinical to first-in-man studies with ^18^F-fludarabine, a novel PET tracer, and the level of evidence concerning its contribution to the treatment of lymphoma malignancies. In the first phase, on the basis of a good rationale, a radiochemical synthesis including purification, characterization, initial formulation, and stability was developed. Then the PET tracer was evaluated with in vitro and in vivo models to assess its biodistribution and estimate radiation dosimetry, and a toxicology study was performed. After a decision was made to translate the tracer to the clinical setting, the process to produce the radiopharmaceutical was transferred to a good-manufacturing environment that ensure final product quality. The next step was a first-in-man trial, a small pilot study for proof of concept and safety. As this novel radiopharmaceutical was proven safe and considered to be of clinical utility larger studies have been undertaken. 

## 3. Lectures

### 3.1. Radio-Iodination Using a C-H Activation Approach (L1)

CaillyThomas^1^,^2^1Normandie Univ, UNICAEN, Centre d’Etudes et de Recherche sur le Médicament de Normandie (CERMN), 14000 Caen, France; thomas.cailly@unicaen.fr2Department of Nuclear Medicine, CHU Côte de Nacre, 14000 Caen, France

Radio-iodinated molecules are used in a wide range of scientific fields, from life sciences to human health applications. These radiotracers are prepared from pre-functionalized chemical precursors, requiring important and time-consuming synthetic efforts. The development of general and easy to implement methods providing access to complex radio-iodinated molecules is of paramount importance to accelerate the discovery process in these fields. Recently transition metal mediated C-H activation has allowed late stage functionalization of complex substrates while using chelating groups already present on these structures. We herein report the palladium mediated C-H radio-iodination of arenes using sodium iodide as the primary isotopic source. This reaction can be performed without chemical know-how in 30 min and has been applied to the synthesis of complex radio-iodinated compounds of biological importance.


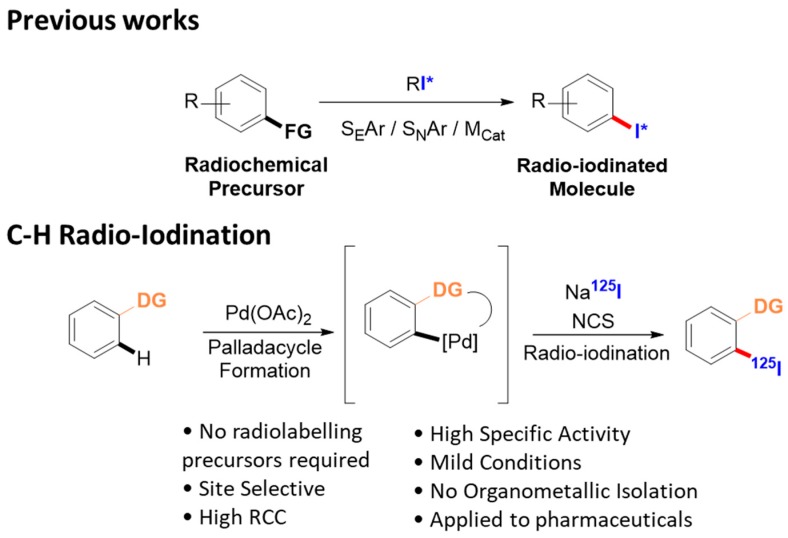


### 3.2. Late-Stage C-H arylation of thiazolo[5,4-f]quinazolin-9(8H)-One Backbone: Synthesis of an Array of Potential Kinase Inhibitors (L2)

FruitCorinneNormandie Univ, UNIROUEN, INSA Rouen, CNRS, COBRA UMR 6014, F-76000 Rouen, France; corinne.fruit@univ-rouen.fr

Our research group was focused on the synthesis of a novel library of thiazolo[5,4-*f*]quinazolin-(on)es as potential kinase inhibitors involved to some extent in Alzheimer’s disease. We previously reported that 4-*N*-protected thiazolo[5,4-*f*]quinazolines displayed single-digit nanomolar IC_50_ values and are among the most potent DYRK1A/1B inhibitors disclosed to date (Nguyen, T. L., et al. *Expert Opin. Ther. Pat.* **2017**, *11*, 1183–1199; Courtadeur, S., et al. *J. Neurochem.* **2015**, *133*, 440–451). Driven by the design of an efficient route allowing a direct transformation of thiazoloquinazolin-4-one derivatives identified as kinase inhibitors (Hédou, D., et al. *Molecules* **2016**, *21*, 578; Hédou, D., et al. *Molecules* **2016**, *21*, 794), the synthesis of an array of C2 and/or C7 arylated compounds was further envisioned. In this context, transition-metal-catalyzed C-C coupling of heteroarene through C-H arylation represents an extremely attractive approach (Rossi, R., et al. *Tetrahedron* **2016**, *72*, 1795–1837; Segawa, Y., et al. *Angew. Chem. Int. Ed.* **2015**, *54*, 66–81; Ackermann, L. *Chem. Rev.* **2011**, *111*, 1315–1345). This methodology has emerged as an important tool for incorporating structural diversity into complex nitrogen containing heterocycles (Aziz, J., et al. *Synthesis* **2017**, *49*, 4562–4585; Fruit, C. *Science* **2016**, *352*, 1277–1278; Wencel-Delord, J., et al. *Nat. Chem.* **2013**, *5*, 369–375). Following our previous study on C-H arylation of quinazolinone skeleton (Laclef, S., et al. *Org. Lett.* **2015**, *17*, 1700–1703; Godeau, J., et al. *Eur. J. Org. Chem*. **2015**, 7705–7717), a selective palladium-catalyzed and copper-assisted direct C-H (hetero)-arylation of thiazolo[5,4-*f*]quinazolin-9(8*H*)-one has been developed with aryl halides as coupling partners under microwave irradiation(Besson, T., et al. *Synthesis* **2016**, *48*, 3879–3889). Electron-deficient heteroarenes are also readily introduced, a notable feature with respect to medicinal agent synthesis (Couly, F., et al. *Synthesis* **2017**, *49*, 4615–4622; Harari, M., et al. *Org. Lett*. **2016**, *18*, 3282–3285).

Differently substituted *N^8^*-protected-aryl-thiazoloquinazolin-9(8*H*)-ones were thereby obtained in a facile manner. This late-stage functionalization of drug candidates allows a streamlined route to compounds with increased novelty, essential in drug discovery.


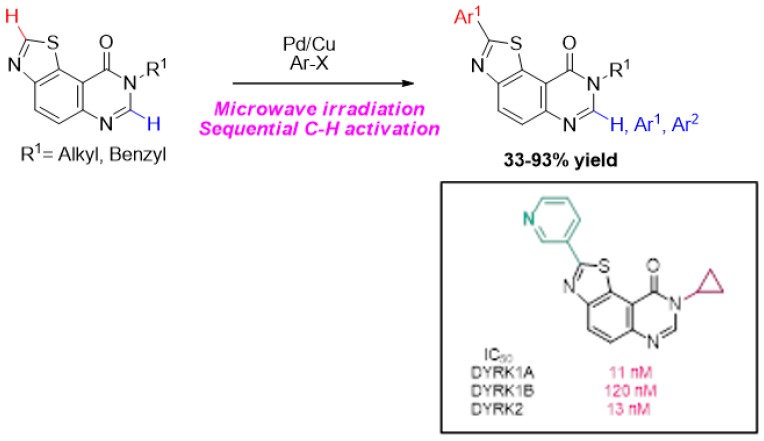


### 3.3. Disrupting the Metabolic Adaptation of Cancer Cells with Small Molecules as a Promising Anticancer Strategy (L3)

FrédérickRaphaëlUniversité Catholique de Louvain (UCL), Medicinal Chemistry Research Group, Louvain Drug Research Institute (LDRI), 73 avenue Mounier, B-1200 Bruxelles, Belgium; raphael.frederick@uclouvain.be

Our current research efforts aims at providing necessary pharmacological or diagnostic tools to detail important evolutionary metabolic strategies allowing cancer cells to cope with fluctuating resource availability and grow. In very recent years, we have been interested in three different axes: the design and study of serine synthetic pathway (SSP) inhibitors, the development of novel original diagnostic tools to study the exchange of lactate between hypoxic/glycolytic cancer cells, and more recently we have started to develop first-in-class lactate dehydrogenase B (LDHB) tetramerization inhibitors. Our last results in this field will be presented (Van Hée, V.F., et al. *Oncotarget* **2017**, *8*, 24415–24428; Ravez, S. et al. *J. Med. Chem.* **2017**, *60*, 1591–1597; Ravez, S. et al. *J. Med. Chem*. **2017**, *60*, 1227–1237; Brisson, L. et al. *Cancer Cell* **2016**, *30*, 418–431).


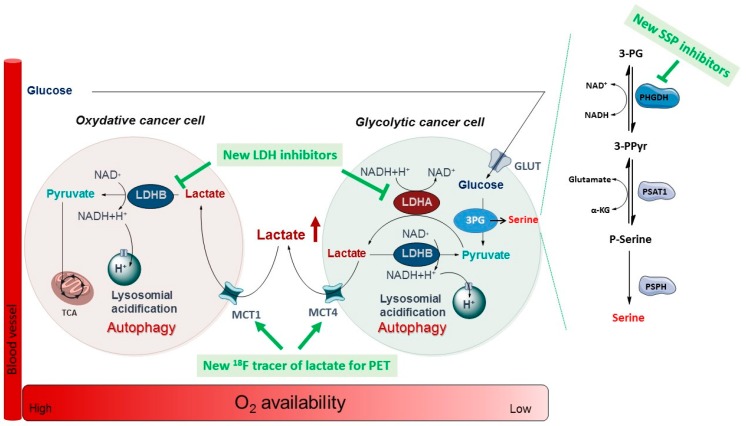


### 3.4. Benzoxepin-Type Selective Estrogen Receptor (ER) Modulators and Downregulators with Subtype-Specific ERα and ERβ Activity (L4)

O’BoyleNiamh M.School of Pharmacy and Pharmaceutical Sciences, Trinity Biomedical Sciences Institute, Trinity College Dublin, 152-160 Pearse St, Dublin 2 D02 R590, Ireland; oboyleni@tcd.ie

The two nuclear estrogen receptors (ERα and ERβ) mediate the biological effects of the estrogen hormones and ERα is an attractive therapeutic target for diseases including breast cancer and osteoporosis. Estrogens are known to have tissue selective effects, and there is considerable interest in the therapeutic use of selective estrogen receptor modulators (SERMs). ERα is an important target for drugs such as tamoxifen and fulvestrant. There is ongoing debate about the role of ERβ in cancer.

Three series of ER-ligands based on the benzoxepin scaffold structure were synthesized—series I containing an acrylic acid, series II with an acrylamide and series III with a saturated carboxylic acid substituent. These compounds were shown to be high affinity ligands for the ER with nanomolar IC_50_ binding values. Series I acrylic acid ligands were generally ERα selective. In particular, compound 13e featuring a phenylpenta-2,4-dienoic acid substituent was shown to be antiproliferative and downregulated ERα and ERβ expression in MCF-7 breast cancer cells. Interestingly, from series III, the phenoxybutyric acid derivative compound 22 was not antiproliferative and selectively downregulated ERβ. A docking study of the benzoxepin ligands was undertaken. Compound 13e is a promising lead for development as a clinically relevant SERD, whilst compound 22 will be a useful experimental probe for helping to elucidate the role of ERβ in cancer cells.


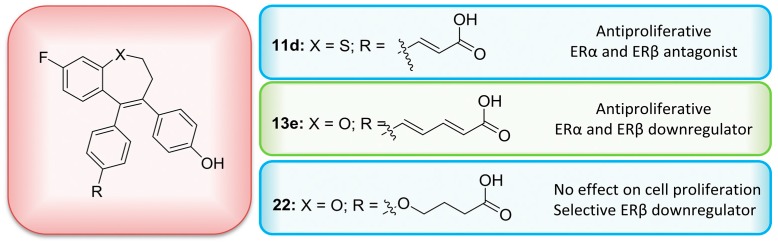


### 3.5. Combining Organofluorine Chemistry and C-H Activation: New Tools to Fluorinated Molecules (L5)

BessetTatianaNormandie Univ, UNIROUEN, INSA Rouen, CNRS, COBRA UMR 6014, F-76000 Rouen, France; tatiana.besset@insa-rouen.fr

Over the last years, the organofluorine research field has known a fast expansion (Liu, H. et al. *Adv. Synth. Catal.* **2013**, *355*, 617–626; Furuya, T., et al. *Nature* **2011**, *473*, 470–477; Besset, T., et al. *Angew. Chem. Int. Ed.* **2012**, *51*, 5048–5050; Wu, X.-F., et al. *Chem. Asian J.* **2012**, *7*, 1744–1754; Landelle, G., et al. *Beilstein J. Org. Chem.* **2013**, *9*, 2476–2536; Besset, T., et al. *Chem. Eur. J.* **2014**, *20*, 16830–16845; Besset, T., et al. *Eur. J. Org. Chem.* **2015**, 2765–2789), which could be explained by the plethora of pharmaceuticals and agrochemicals containing at least one fluorine atom (Wang, J., et al. *Chem. Rev.* **2014**, *114*, 2432–2506; Ilardi, E. A., et al. *J. Med. Chem.* **2014**, *57*, 2832–2842; Gillis, E. P., et al. *J. Med. Chem*. **2015**, *58*, 8315–8359; Purser, S., et al. *Chem. Soc. Rev.* **2008**, *37*, 320–330; Landelle, G., et al. *Curr. Top. Med. Chem.* **2014**, *14*, 941–951). Consequently, a large panel of fluorinated groups (F, CF_3_, SCF_3_, CF_2_R…) is available and their introduction onto molecules can be realized by means of various transformations. Besides, transition metal catalyzed direct C-H bond functionalization has witnessed tremendous progress over the last decade allowing new retrosynthetic disconnections and innovative approaches. The combination of organofluorine chemistry and the transition metal catalyzed C-H bond functionalization is really appealing and is considered as a powerful synthetic tool. However, the introduction of fluorine-containing groups on versatile alkenes as well as aliphatic derivatives by direct C-H bond functionalization is still scarce. To take up this challenge, we have developed innovative methodologies to access these important fluorinated building blocks. Transition metal catalyzed trifluoromethylation (Besset, T., et al. *J. Org. Chem.* **2014**, *79*, 413–418) and trifluoromethylthiolation (Xiong, H.-Y., et al. *J. Org. Chem.* **2015**, *80*, 4204–4212; Zhao, Q., et al. *Org. Lett.* **2017**, *19*, 5106–5109) of vinylic and aliphatic amides were particularly studied. Besides, a special attention was paid to the development of modern strategies in organofluorine chemistry with a special focus on emergent fluorinated groups and the design of original electrophilic reagents (Xiong, H.-Y., et al. *Org. Chem. Front.* **2016**, *3*, 620–624; Xiong, H.-Y., et al. *Angew. Chem. Int. Ed.* **2016**, *55*, 13490–13494).


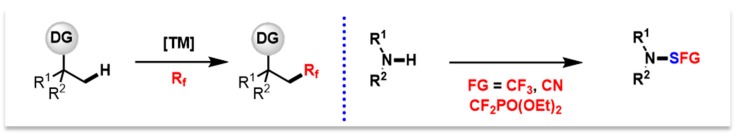


### 3.6. Molecular Interrogation of Multiple Signalling States of the β_1_-Adrenoceptor (L9)

MistryShailesh N.^1^*BakerJillian G.^2^1School of Pharmacy, Centre for Biomolecular Sciences, University of Nottingham, Nottingham NG7 2RD, UK2Cell Signalling, The University of Nottingham Medical School, Queen’s Medical Centre, Nottingham NG7 2UH, UK*Correspondence: shailesh.mistry@nottingham.ac.uk

The β_1_-adrenoceptor (β_1_-AR) is a predominantly cardiac G protein-coupled receptor (GPCR) and regulates both the rate and force of heartbeat. β_1_-AR blockade is important in managing symptoms and prolonging life in heart failure and ischaemic heart disease, by blocking the actions of endogenous catecholamine agonists—epinephrine and norepinephrine. Conventionally it was believed that β_1_-AR activation occurred solely through agonist binding to the catecholamine binding site and stabilisation of an active receptor conformation, however β-blockers (β_1_-AR antagonists) such as CGP12177 have been found to exhibit an unusual behaviour (Kaumann, A. J., et al. *Pharmacol. Therapeut.* **2008**, *118*, 303–336). It is now well established that the β_1_-AR exists in at least two agonist signalling states. A ‘primary conformation’, which is activated by endogenous catecholamines and competitively blocked by β-blockers with high binding affinity and, a ‘secondary conformation’, through which some ligands (e.g., CGP12177, pindolol, alprenolol) can generate agonist responses that are relatively resistant to β-blocker antagonism (Baker, J. G., et al. *Mol. Pharmacol.* **2003**, *63*, 1312–1321).


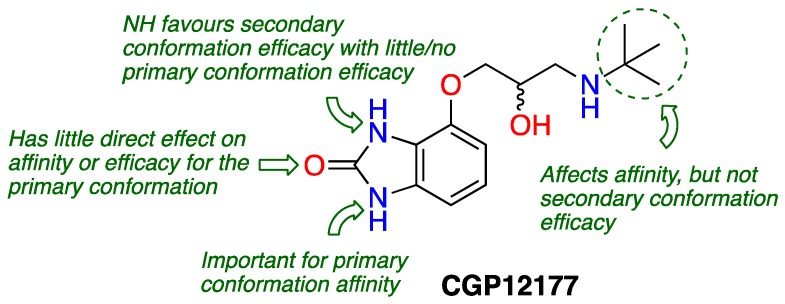


Of concern, some β-blockers that exhibit this activity (e.g., carvedilol) and are used in the clinic reach plasma concentrations in patients that are sufficient to enable secondary conformation-mediated signalling. In order to understand this phenomenon further it is essential to elucidate the specific ligand-protein interactions and ligand structural features that are responsible for this behaviour. This presentation will summarise our findings to date of an ongoing ligand-based SAR study which started with two pharmacologically unusual β-blockers – CGP12177 and alprenolol.

### 3.7. Epigenetic Reactivation: HDAC and LSD1 Inhibitors as Anticancer Agents (L10)

GanesanA.School of Pharmacy, University of East Anglia, Norwich Research Park, Norwich NR4 7TJ, UK; a.ganesan@uea.ac.uk

The activation or silencing of genes in nucleosomes is regulated by structural modifications of the DNA double helix and the N-terminal tails of histone proteins. Two of the major histone modifications involve methylation or acetylation of lysine residues which are then removed by deacetylase (HDAC) and demethylase (KDM) enzymes. Both the HDACs and KDMs are important drug discovery targets, with five recently approved agents for the treatment of haematological cancers and multiple other candidates in clinical trials. The presentation will review the state of the art for HDAC and KDM inhibitors with illustrative examples based on natural products and synthetic scaffolds from our group (Lecointre, B., et al. *Phil. Trans. R. Soc. B* **2018**, *373*, 20170364; Conforti, F. et al. *Oncotarget* **2017**, *8*, 48737–48754; Borrello, M. T. et al. *Bioorg. Med. Chem. Lett*. **2017**, *27*, 2099–2101; Liu, Q. et al. *RSC Adv.* **2015**, *5*, 1109–1112; Tortorici, M., et al. *ACS Chem. Biol*. **2013**, *8*, 1677–1682).


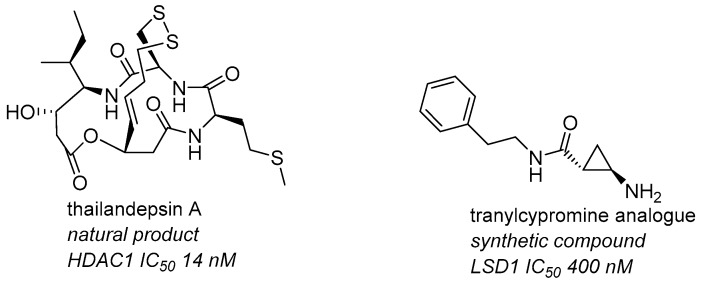


### 3.8. New Azole Antifungals with Fused Pyrimidinone and Triazinone Derivatives (L11)

LogéCédric^1^*MontoirDavid^1^GuillonRémi^1^TonnerreAlain^1^PicotCarine^2^GazzolaSophie^2^PagniezFabrice^2^Le PapePatrice^2^1Department of Medicinal Chemistry, University of Nantes, F-44200 Nantes, France2Department of Parasitology and Medical Mycology, University of Nantes, F-44200 Nantes, France*Correspondence: cedric.loge@univ-nantes.fr

Azoles (fluconazole, itraconazole, voriconazole, posaconazole) are important drugs for the treatment of invasive fungal infections (IFIs). Unfortunately, their use as first intention has led to resistance. Besides azole antifungal agents, alternative classes include polyenes such as amphotericine B and its lipidic forms, as well as the echinocandines. However, these alternative antifungal agents are generally costly and not suitable for oral administration. Thus, there is a continued interest in developing azole antifungals in particular those able to circumvent resistance phenomenon’s which can be attributed either to mutations in *ERG11* gene encoding the cytochrome P450 14α-demethylase (CYP51), or to an overexpression of genes encoding membrane transport proteins (CDR1, CDR2, and MDR1). Recently, isavuconazole, a new extended-spectrum triazole was also approved for the treatment of invasive aspergillosis and mucormycosis, an order of molds that most antifungals (voriconazole included) do not treat.

We have already reported the synthesis of a novel antifungal agent bearing a thiazoloquinazolinone scaffold (Guillon, R. et al. *ACS Med. Chem. Lett.* **2013**, *4*, 288–292). Taking into account that this compound has showed in vivo antifungal efficacy in a mice model of systemic candidiasis, we decided to synthesize new analogues with a more easily accessible pyrrolotriazinone or imidazopyrimidinone scaffolds (Logé, C., et al. PCT WO 2017/021178 A1). These compounds exhibit in vitro activity against pathogenic *Candida* species (fluconazole-susceptible and fluconazole-resistant) and promising activity against both *Aspergillus fumigatus* strains and a *Rhizopus oryzae* strain, the most prevalent agent of mucormycosis. Our lead compounds displayed also in vivo efficacy against three lethal systemic infections caused by *C. albicans*.

### 3.9. Pyridoclax and Its Derivatives Directly Inhibit Mcl-1 and Exert Potent Antitumor Effects on Ovarian Cancers In Vitro and In Vivo (L13)

Voisin-ChiretAnne SophieNormandie Univ, UNICAEN, Centre d’Etudes et de Recherche sur le Médicament de Normandie (CERMN), 14000 Caen, France; anne-sophie.voisin@unicaen.fr

Protein-protein interactions (PPIs) are attractive targets because they control numerous cellular processes. In oncology, apoptosis regulating Bcl-2 family proteins are of particular interest. Apoptotic cell death is controlled via PPIs between the anti-apoptotic proteins hydrophobic groove and the pro-apoptotic proteins BH3 domain. In ovarian carcinoma, it has been previously demonstrated that Bcl-x_L_ and Mcl-1 cooperate to protect tumor cells against apoptosis.

If clinically relevant pharmacologic inhibition of Bcl-x_L_ is available using ABT-263 (Navitoclax), selective direct inhibition of Mcl-1 remains problematic. In this context, our teams have designed and synthesized small compounds based on a pyridyl scaffold, named oligopyridines, which potentially target the Mcl-1 hydrophobic binding pocket. We demonstrated that the lead of the first generation of oligopyridines, named *Pyridoclax*, interacts directly with Mcl-1, releases its pro-apoptotic partners Bim and Bak and induces massive apoptosis at 25 µM concentration in combination with anti-Bcl-x_L_ strategies in chemoresistant ovarian cancer cell lines.

In order to improve its biological activity, we evaluated the cytotoxic effects of a second generation of oligopyridines derived from the Pyridoclax. This allowed us to identify the *MR31367*, one of the most potent oligopyridines that shows a stronger pro-apoptotic activity in association with to Bcl-x_L_-targeting strategies in ovarian cancer cell lines. Further characterization showed that this derivative binds Mcl-1 and releases Bim and Bak from it, leading to Bak-mediated apoptosis.

Overall, these results open up interesting perspectives for the clinical use of Mcl-1 inhibitors as single agent or in combination with anticancer drugs to improve the clinical management of ovarian cancers.

## 4. Young Researcher Communications

### 4.1. Crystallization Additives, Not so Useless in In-Silico Kinase Research (YRC01)

FoghaJade^1^DiharceJulien^1^ObledAlan^2^BonnetPascal^1^*1Institut de Chimie Organique et Analytique—ICOA UMR7311, Université d’Orléans, Pôle de Chimie, rue de Chartres, 45000 Orléans, France2School of chemistry, University of St Andrews, North Haugh, St Andrews, Fife KY16 9ST, UK*Correspondence: pascal.bonnet@univ-orleans.fr

Generally, drug design processes referred to the modulation of a therapeutic target through the binding of a ligand into its active or orthosteric site. In contrary to this direct modulation, another concept progressively aroused a great interest in therapeutic research: allostery (Cominetti, M.D.D., et al. *Org. Biomol. Chem.* **2016**, *14*, 10161–10164). Indeed, defined allosteric sites open the way to the conception of allosteric ligands, which present some advantages such as greater specificity (Peracchi, A., et al. *Biochim. Biophys. Acta—Proteins Proteomics* **2011**, *1814*, 922–933). In this study, we proposed an innovative way to identify allosteric sites, based on the study of the distribution of crystallization additives, used to stabilize proteins during the crystallization process.


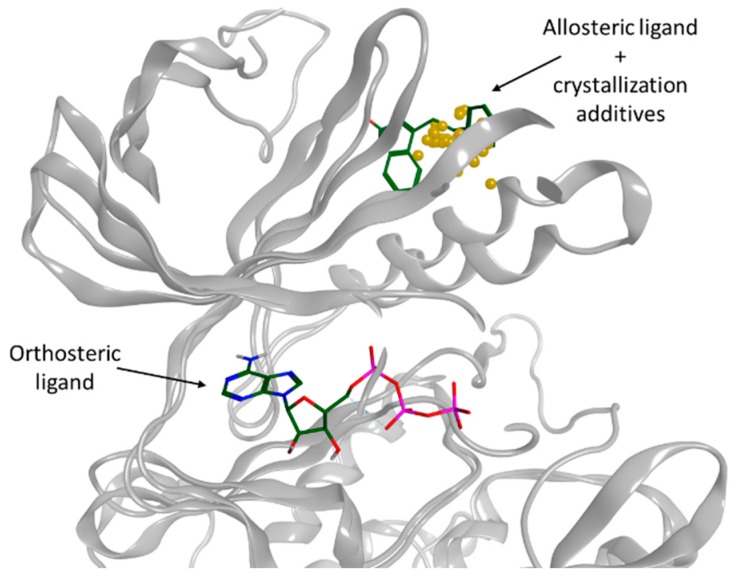


Orthosteric, allosteric ligands and centroids of crystallographic additives represented in aligned protein kinases (PDB ID: 3K5V(Zhang, et al. *Nature **2010***, *463 (7280)*, 501–506), 3HRF (Hindie, et al. *Nat. Chem. Biol.* **2009**, *5 (10)*, 758–764). 

Density and clustering analysis of those compounds, applied on protein kinase family, revealed that the distribution of those compounds is not randomly spread around the structure, but the ligands have the tendency to aggregate near important sites. Interestingly, this original strategy allows the identification of defined allosteric sites. Moreover, crystallization additives could detect new allosteric sites in kinases and could be extend to other protein families.

### 4.2. Fluorine-Dependent Regioselective Opening of Oxetan-3-ylidene Derivatives (YRC02)

FontenelleClément*LaporteRomainPfundEmmanuelLequeuxThierry*Laboratoire de Chimie Moléculaire et Thioorganique, ENSICAEN, UNICAEN, CNRS UMR 6507, 6 Bd du Maréchal Juin, 14050 Caen, France*Correspondence: clement.fontenelle@ensicaen.fr (C.F.); thierry.lequeux@ensicaen.fr (T.L.)

Four membered cycles are important strained structures in particular azetidines and oxetanes have attracted much attention in drug discovery (Carreira, E. M., et al. *Chem. Rev.* **2014**, *114*, 8257–8322; Burkhard, J. A., et al. *Angew. Chem. Int. Ed.* **2010**, *49*, 9052–9067). They have been introduced to modify the pharmacokinetic properties of new drugs but they can also be opened selectively leading to molecules with various and all different substituents (Hoste, J., et al. *Bull. Soc. Chim. Belg.* **1949**, *58*, 157–166; Burkhard, J. A., et al. *Org. Lett.* **2008**, *10*, 3525–3526; Yadav, J. S., et al. *Org. Lett.* **2014**, *16*, 836–839). In addition, fluorine atom introduction is well known to modify the electronic properties of the neighbouring functions. In connection with our recent programme on the synthesis of antibiotics, we explored the influence of the fluorine atom on the regioselectivity of the ring-opening reaction of oxetan-3-ylidene derivatives to obtain *trans*-fluorobutenyl structures **2** as nucleoside mimics. Compound **3** is of particular interest as analogue of known *Mycobacterium Tuberculosis* FDTS inhibitors (Herdewijn P., et al. *Chem. Med. Chem.* **2013**, *8*, 1373–1383).


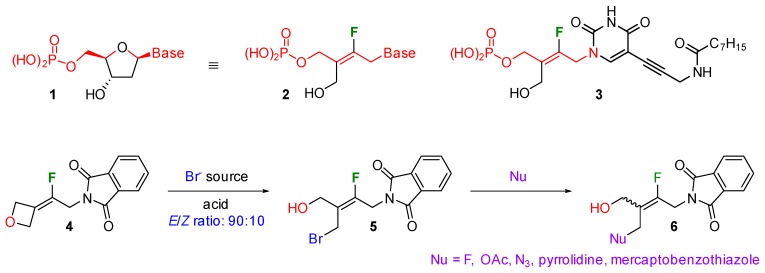


Excellent and opposite regioselectivities were observed for the phthalimidomethyl derivatives depending on the absence or presence of the fluorine atom. The bromide could readily be substituted by a number of nucleophiles thereby providing an interesting diversity. Other substrates bearing a benzyloxymethyl or an ester group were investigated and demonstrated similar reactivities. Particularly in the case of the ester, both *E* and *Z* regioisomers with ratios greater than 84:16 could be obtained depending on the conditions used. This communication decribes these results and will aim at proposing a mechanism.

**Acknowledgments:** This work was supported by the Excellence Laboratory LabEx SYNORG (ANR-11-LABX-0029), the Conseil Régional de Normandie, and the European FEDER fundings.

### 4.3. Re-Designing Ecotoxic Drugs: From Environmental Persistence to Programmed Inactivation through Self-Immolation (YRC03)

Abellán-FlosMartaRascolEstelleLabruèreRaphaël*ICMMO, University of Paris-Saclay, 91405 Orsay, France*Correspondence: raphael.labruere@u-psud.fr

Active pharmaceutical ingredients (APIs) and their metabolites (mAPIs) are commonly found in the environment since wastewater treatment plants (WWTP) are not generally prepared to deal with them. The toxic effects of APIs/mAPIs on organisms caused by their intrinsic properties, wide variety, presence as a mixture and chronic exposure urges the scientific community to consider the entire life cycle of drugs and environmental impact (Kümmerer, K. *Green Chem.* **2007**, *9*, 899–907).

Our project is based on the structural modification of already marketed drugs for programming them towards self-immolation after having fulfilled their therapeutic purpose. Indeed, once excreted, our rationally designed eco-drugs will undergo particular modifications in the biomass by predictable metabolism, which will trigger their self-disassembly, consequently losing their original activity and toxicity (Alouane, A., et al. *Angew. Chem. Int. Ed.* **2015**, *54*, 7492–7509).


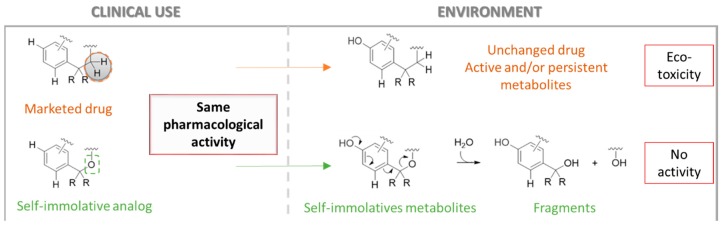


Methotrexate (MTX), one of the most widely prescribed antitumor agents worldwide since the 40’s, was initially selected for its ability to accommodate a self-immolative structure via straightforward modification. MTX and its major metabolite, 7-OH-MTX, were detected in hospital effluents and WWTP effluents (Besse, J. P., et al. J. *Environ. Int.* **2012**, *39*, 73–86), leading to potential toxic effects against wildlife (Białk-Bielińska, A., et al. *Chemosphere* **2017**, *189*, 689–698). An analogue bearing the proper structural modification and displaying a similar in vivo activity has been synthesized (Nair, M. G., et al. *J. Med. Chem.* **1981**, *24*, 1068–1073). In our project, the better eco-compatibility of this compound is investigated in comparison to the methotrexate. Hence, metabolism in biological media and environmental transformation are under evaluation.

**Acknowledgments:** This work was funded by the Laboratory of Excellence in Research on Medication and Innovative Therapeutics (LERMIT) supported by a grant ANR-16-CE34-0001 titled “EDIFIS - Ecodesign of drugs containing a self-immolative scaffold”.

### 4.4. In Vitro Evaluation of ROS-Activatable Anticancer Boronate Prodrugs of doxorubicin (YRC04)

SkarbekCharles*SerraSilviaRascolEstelleLabruèreRaphaëlInstitut de Chimie Moléculaire et des Matériaux d’Orsay (ICMMO), CNRS, Univ Paris Sud, Université Paris-Saclay, 15 rue Georges Clemenceau, 91405 Orsay Cedex, France*Correspondence: charles.skarbek@u-psud.fr

Pharmaceutical industries and public research centers have made oncology one of their priorities. Many drugs have been introduced on the market in order to treat cancer, however, many still suffer from a lack of selectivity for tumor cells over normal cells resulting in insufficient drug concentrations in tumors, systemic toxicity and appearance of drug-resistant tumor cells. To circumvent these drawbacks, a relevant strategy relying on the development of prodrugs designed to be activated after an enzymatic or a chemical reaction near the site of action allowing a specific release of the drug at its target site has rose (Monika A., et al., *Eur. J. Med. Chem.* **2017**, *129*, 53–71.). Among the different metabolic pathways, the activation by reactive oxygen species (ROS), such as hydrogen peroxide (H2O2), appears particularly interesting and recent studies have shown that this property could be exploited for therapeutic benefit (Trachootham D., et al., *Nat. Rev. Drug Discov.* **2009**, *8*, 579–591). Since the cleavage of aryl boronic acids and their ester derivatives takes place in presence of H_2_O_2_, we studied the design and development of new anticancer prodrugs consisting in the coupling of a pinacol boronate ester (trigger unit) to doxorubicin (active entity). 


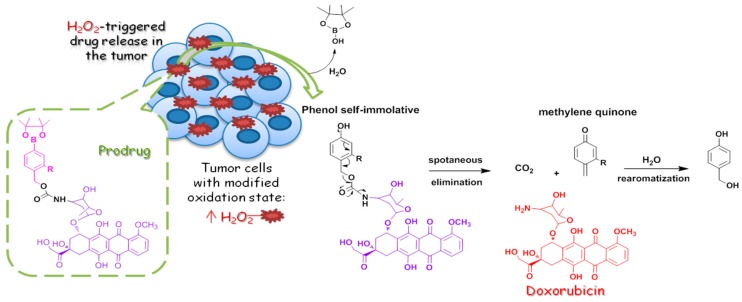


Benzeneboronates, in which the oxidation of the carbon-boron bond allows the formation of an electron donor alcohol group, act as quinone methide-based self-immolative spacers, leading to the release of the active drug after an electronic delocalization within the aromatic nucleus. A panel of prodrugs has been designed with several kind of benzeneboronates rings on the basis of established structure-activity relationships (Cao S., et al., *Chem. Eur. J*. **2013**, *19*, 9050–9058.; Alouane A., et al., *Angew. Chem. Int. Ed*. **2015**, *54*, 7492–7509). Indeed, a short self-immolation time will allow to limit the lifetime of the self-immolative phenol and, thus, limit its diffusion outside the tumor cells. A prosensor has been designed and used on a panel of tumor cell lines to investigate their ability to produce H_2_O_2_. Then, the designed prodrugs were investigated in vitro to determine their IC50 compared to the parent drug.

**Acknowledgments:** This work has been supported by the Fondation ARC pour la recherche sur le cancer.

## 5. Flash Communications

### 5.1. Phenotype Characterization and Mechanism Determination of TMEM45A Protein (FC01)

Dal MasoThomas^1^*MarxSebastien^1^MichielsCarine^2^WoutersJohan^1^Le CalvéBenjamin^2^1Department of Chemistry, University of Namur, 5000 Namur, Belgium2Department of Biology, University of Namur, 5000 Namur, Belgium*Correspondence: thomas.dalmaso@unamur.be

More and more cancers display resistance to chemotherapeutic drugs and are linked to well- known factors such as tumor microenvironment. For example, hypoxia has been shown to favour tumor growth and to be involved in the drug resistance of cancer cells. During hypoxia, cell metabolism switches for adapting to environment modifications. Flamant et al. highlighted a new transmembrane protein, TMEM45A, which could be involved in the resistance of cancer cell in hypoxic conditions (Flamant, L. et al. *BMC Cancer* **2012**, *12*, 391). It is known that the silencing of TMEM45A in human breast cancer cells (MDA-MB-231) incubated in the presence of etoposide or taxol under hypoxia become sensitive to cell death induced by these chemotherapeutic molecules. Sun et al. have already shown that the knockdown of this protein has an impact on invasion, migration and proliferation of glioma cells (T98G) (Rebucci, M. et al. *Biochem. Pharmacol*, **2013**, *85*, 1219–1226; Sun, W. et al. *Int. J. Clin. Exp. Pathol.* **2015**, *8*, 12657–12667).

Until now, various phenotypes were observed and associated to TMEM45A expression. However, the structure, function and biological pathway of this protein are unknown. This work aims to unravel the mechanism of actions and functions by determining partners of TMEM45A using immunoprecipitation assays coupling to mass spectrometry. Moreover, the phenotype was studied using shRNA technology in MDA-MB-231 and T98G cells.

**Acknowledgments:** Thomas Dal Maso is grateful for financial support from the Fonds pour la Recherche Scientifique (FNRS). This work is supported by FRIA Grant.

### 5.2. Conception and Synthesis of Novel MT5-MMP Inhibitors for Alzheimer’s Disease (FC02)

ZipfelPauline^1^*LalutJulien^1^GiovanniniJohanna^1^SuzannePeggy^1^LepailleurAlban^1^BureauRonan^1^BarangerKevin^2^KhrestchatiskyMichel^2^RiveraSantiago^2^RochaisChristophe^1^DallemagnePatrick^1^1Centre d’Etudes et de Recherche sur le Médicament de Normandie (CERMN), Normandie Univ, UNICAEN, 14000 Caen, France2Institute of Neuropathophysiology (INP), UMR7051, CNRS, Aix Marseille Université, 13331 Marseille Cedex 03, France*Correspondence: pauline.zipfel@unicaen.fr

In 2017, over 46 million people were living with dementia worldwide and Alzheimer’s disease (AD) is the most common form of dementia in France, with about 850,000 cases. AD is a neurodegenerative and incurable brain disorder; only treatments for symptoms are available at this time. Because of the heavy economic and societal impact, there is an urgent need to find new treatments that target the molecular causes of neuronal cell death.

Two abnormal structures in the brain called β-amyloid (Aβ)-containing plaques and neurofibrillary tangles are considered as two of the main features of AD. In this context, several studies support the hypothesis that alterations in the processing of β-amyloid precursor protein (APP), resulting in the accumulation of Aβ and other proteolytic products contributes to AD pathogenesis. Thus, current research focuses on the enzymes involved in the amyloid cascade such as α-, β-, and γ-secretase. However, recent studies have revealed the existence of another physiological APP processing pathway, mediated by a novel AD-related enzyme, membrane-type 5-matrix metalloproteinase (MT5-MMP), that can process APP and promote Aβ and CTFβ accumulation, as well as the inflammatory process in AD transgenic mice (Baranger, K. et al. *Cell. Mol. Life Sci.* **2016**, *73*, 217–236; Baranger, K. et al. *J. Neuroinflammation* **2016**, *13*, 167; Baranger et al. *Front. Mol. Neurosci.* **2017**, *9*, 1–17). Moreover, MT5-MMP can cleave APP upstream from the β-secretase cleavage site (the so called η-cleavage site) (Ahmad, M. et al. *J. Biochem.* **2006**, *139*, 517–526) and release a *N*-terminally elongated Aβ fragment (Aη-α), which appears to be synaptotoxic (Willem, M. et al. *Nature* **2015**, *526*, 443–447).

We aim to design and synthesize the first MT5-MMP inhibitors through an interdisciplinary approach including molecular modelling, medicinal chemistry and biology. Starting from a hit compound identified by screening of CERMN’s chemical library, we are now investigating the pharmacomodulations on that scaffold to gain in affinity and selectivity for MT5-MMP.


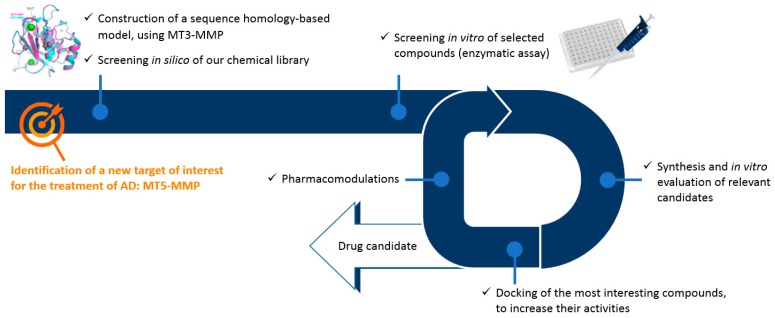


### 5.3. Multicomponent Ugi-Synthesis and Biological Evaluation of New Multitarget Directed Small Molecules for Alzheimer’s Disease Therapy (FC04)

Pachón-AngonaIrène*IsmailiLhassaneNeuroscience EA481, Université de Bourgogne Franche-Comté, 19 rue Ambroise Paré, 25000 Besançon, France*Correspondence: pachon.angona.irene@gmail.com

Alzheimer’s disease (AD) is a multifactorial and fatal neurodegenerative disorder characterized by decline of cholinergic function, deregulation of other neurotransmitter systems, beta-amyloid (Aβ) fibril aggregation and neurofibrillary tangles deposition (León, R., et al. *Med. Res. Rev.* **2013**, *33*, 139–189). Due to this complex nature of AD and the involvement of a number of relevant biological systems in AD progression, multitarget directed ligands (MTDL) may enable therapeutic efficacy. Accordingly, compounds possessing, besides anticholinergic activity and Aβ-aggregation inhibition properties, biometal chelating, nitric oxide releasing properties, antioxidant capacity, beta-secretase and monoamine oxidase inhibition power, as well as serotonin and sigma receptor modulation capacities have been developed.

In this context, we describe here new MTDL synthesized by the multicomponent Ugi reaction (Pachón-Angona I., et al., *J. Enzyme Inhib. Med. Chem.* **2018**, 34, 479–489). The new hybrids result from the juxtaposition of different pharmacophoric groups, such as N-benzylpiperidine, a well-known functional motif present in ChE inhibitors, melatonin, a natural product showing broad-spectrum antioxidant and free radical scavenger activity, and a chromone moiety, as an antioxidant and MAO inhibitor.


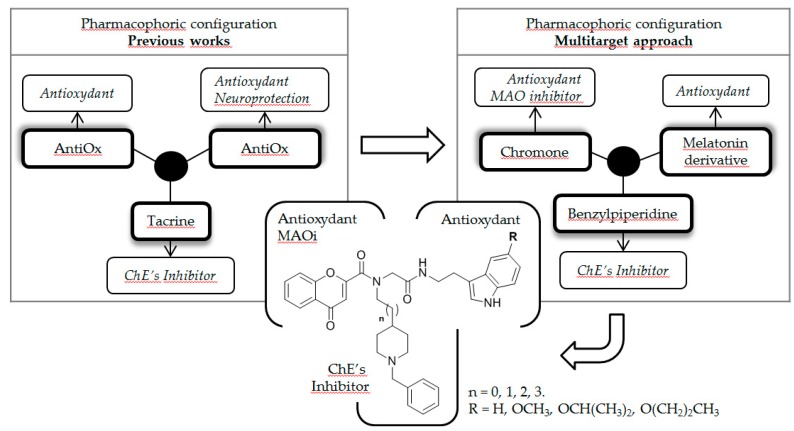


Accordingly, the new MTDL showed a strong antioxidant power, *Ee*AChE, *eq*BuChE and hMAO-A/B inhibitory activities, and neuroprotective capacity in SH-SY5Y neuronal cells against toxics insults, such as Oligomycin/Rotenone, Aβ_1-35_ and okadaic acid.

### 5.4. Synthesis of Piperazine-Based Siderophores to Design Broad Spectrum Antibiotics (FC06)

LoupiasPauline^1^*Dassonville-KlimptAlexandra^1^LohouElodie^1^TaudonNicolas^2^SonnetPascal^1^1AGIR, UFR de pharmacie, Université de Picardie Jules Verne, 1 rue des Louvels, 80000 Amiens, France2Unité de Toxicologie Analytique, Institut de Recherche Biomédicales des Armées, 91223 Brétigny-sur-Orge, France*Correspondence: pauline.loupias@etud.u-piardie.fr

Resistance to antibiotics is an emerging phenomenon, fast becoming a major medical problem. The resistance of Gram-negative bacteria such as *Pseudomonas aeruginosa* and the *Burkholderia* group to conventional antibiotics leads to therapeutic failure and requires new antibiotic therapies. The use of iron transport systems is a promising strategy to overcome this phenomenon. These TonB-dependent receptors, essential for the survival of microorganisms, allow specific recognition of ferric siderophore complexes in order to transport iron within bacteria (Miethke, M. et al., *Microbiol. Mol. Biol. Rev*. **2007**, *71*, 413–451). Bacteria, according to their kind, express different types of receptors that allow them to recognize their endogenous siderophores but also xenosiderophores. *Pseudomonas aeruginosa* and *Burkholderia* pseudomallei in particular possess FptA receptors allowing the recognition of pyochelin (Butt A.T., et al. *Front. Cell. Infect. Microbiol.* **2017**, 7).

These specific systems may allow the introduction of antibacterial agents by forming antibiotic-siderophore conjugates or toxic complexes such as gallium complexes, in the bacteria to kill it. Siderophores have three types of chelating function: catechols, hydroxamates and hydroxy-carboxylates. Previous work in the laboratory has shown that piperazine 1,4-dicatechol structures (MPPS0225) could be recognized by *Pseudomonas aeruginosa* strains. In order to further investigate this piperazine platform, we have synthesized iron chelators bearing 3-hydroxypyridin-4-ones and 1,3-dihydroxypyridin-4-one ligands. At the same time, we were interested in the synthesis of a more complex 2,5-dioxopiperazine platform, part of the rhodotorulic acid (RA), a natural siderophore produced by *Rhodotorula pilimanae* showing an interesting iron affinity (pFe = 21,8). Two RA synthesis strategies will be developed as well as the corresponding 3,6-disubstituted analogs. Through the synthesis of these chelators, we would like to study the influence, on the complexation of iron, the nitrogenous platform (piperazine or dioxopiperazine), the presence of stereogenic centers (3,6-disubstituted dioxopiperazine vs piperazines 1,4 -disubstituted) and the nature of the iron ligands (hydroxypyridinone vs catechol). An evaluation of the siderophore-like potential and a measurement of the complexing force of these will be carried out.


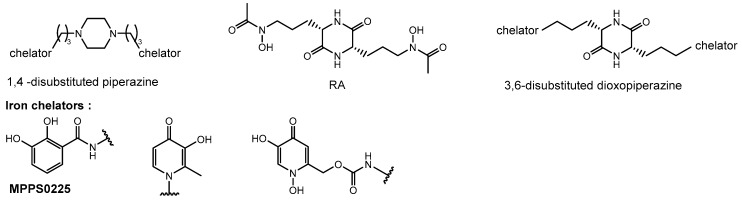


**Acknowledgments:** This work was funded by the DGA and the Haut de France region.

### 5.5. Docking Studies of Melatonergic and Serotonergic Multi-Target Directed Ligands of Potential Interest for Alzheimer’s Disease (FC07)

ThirumaranSangeetha-Laura*XiaoFengLemaîtreStéphaneBureauRonanRaultSylvainLepailleurAlbanRochaisChristopheCentre d’Etudes et de Recherche sur le Médicament de Normandie (CERMN), Normandie Univ, UNICAEN, 14000 Caen, France*Correspondence: sangeetha-laura.thirumaran@unicaen.fr

Alzheimer’s disease (AD) is the most common form of dementia, affecting millions of patients worldwide, for which the current treatments produce only symptomatic benefits. Among the biological targets implied in the physiopathology, and especially among the G-protein coupled receptors (GPCRs), melatonergic MT1 and MT2 and serotonergic 5-HT_2c_ receptors present a growing interest. Modulations of these receptors have been proved to promote the non-amyloidogenic cleavage of Amyloid Protein Precursor (APP) and to ameliorate the symptoms through several actions such as anti-oxidant effect and regulation of the transmission of other neurotransmitters (Shukla, M., et al., *Curr. Neuropharmacol.* **2017**, *15*, 1010–1031; Švob Štrac, D., et al., *Transl. Neurosci.* **2016**, *7*, 35–49).

As AD is a multifactorial disorder, a simultaneous action on these two types of receptors with Multi-Target Directed Ligands (MTDLs) could represent a novel therapeutic approach. To this aim, we screened our chemical library in order to identify both potent MT1 and MT2 receptors agonists and 5-HT_2c_ receptors antagonists. We performed docking studies of the selected molecules into homology models of MT1 and MT2 receptors and into the crystal structure of 5-HT_2c_ receptors (Peng, Y., et al. *Cell*. **2018**, *172*, 719–730). The resulting ligand-receptor interactions are in agreement with the literature and allow to understand the polypharmacological profile of this promising new series of compounds (Pala, D., et al., *Int. J. Mol. Sci*. **2013**, *14*, 8093–8121; Renault, N. in Di Giovanni, G.; Esposito, E.; Di Matteo V. (Editors), *5HT_2C_ Receptors in the Pathophysiology of CNS Disease*, **2011**, 97–127).


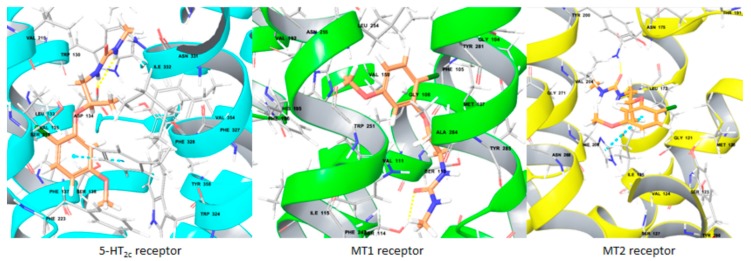


### 5.6. Versatile Approach to 5,6-Member Ring Cephalosporin Derivatives as Promising P2X7R Ligands via Morin Rearrangement (FC08)

DantonFanny*OthmanMohamedLawsonAta MartinDaichAdamNormandie Univ, UNILEHAVRE, URCOM, CNRS FR-3038, Le Havre, France Université Le Havre Normandie, 25 rue Philippe Lebon, BP. 1123, 76063 Le Havre, France*Correspondence: fanny.danton@univ-lehavre.fr

The high P2X7R receptor expression in immune cells suggests its considerable role in numerous diseases such as cancer and neurodegenerative diseases. Previous studies have already proved the anti-inflammatory, analgesic and in some cases anticancer properties of selective P2X7R antagonists (Baudelet, D., et al. *Curr. Med. Chem.* **2015**, *22*, 713–729).

Among the different chemical series developed by the major pharmaceutical companies, some lead compounds underwent to clinical trials mainly for the treatment of inflammatory diseases.^1^ GSK products of types **1** and **2** (Chambers, L. J. et al., WO 2008003697, **2008**, *Chem. Abstr.* **2008**, *148*, 145026) mentioned in the Figure are allosteric modulator of P2X7R receptor. The latter were proposed for the first time in 2008 by GlaxoSmithKline for the treatment of chronic inflammatory.

The aim of our work is to elaborate new molecules able to interact with P2X7R receptors. The design of our compound series was done on the basis of the GSK lead compounds **1** and **2**. Indeed, the 2-amido-lactam “-**C-CO-NH-C**-” sequence found in compounds **1** and **2** was considered as the key fragment responsible of such hypothetic activity. Therefore, we envisioned to insert such moiety in our series. As highlighted in the Figure, a skeleton containing a polycyclic *N*,*X*-acetal backbone (**I, II**; X = S, O, N), fused 1,4-thiazine **III** and 1,4-diazine **IV** bearing the expected 2-amidolactam fragment were selected to ensure a structure-activity relationship (SAR) study pivotal for further biological activities.


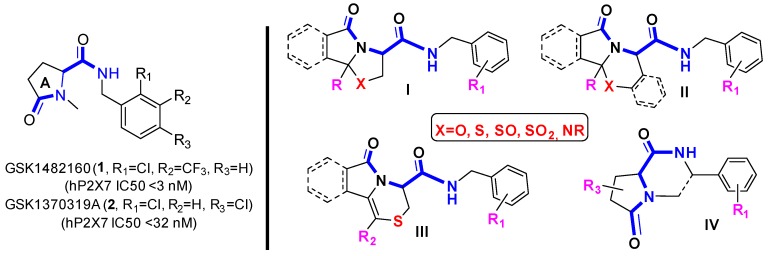


In this line, the expected *N*,*X*-acetals **I, II** were synthetized by using a modified standard Meyer’s cyclocondensation (Groaning, M. D.; Meyers, A. I. *Tetrahedron* **2000**, *56*, 9843–9873). Then an oxidation step followed by a Morin rearrangement provided the compounds **III** starting from **I** and **II**. During these investigations, the experimental conditions for the sulfoxides intermediates as well as the Morin rearrangement were investigated and optimized.

### 5.7. Design, Synthesis and Biological Evaluation of Dibenzofuran Derivatives Inspired from Cercosporamide as Kinase Inhibitors (FC09)

DaoViet Hung^1^Ourliac-GarnierIsabelle^2^BazinMarc-Antoine^1^BaratteBlandine^3^RuchaudSandrine^3^BachStéphane^3^Le PapePatrice^2^MarchandPascal^1^*1Department of Medicinal Chemistry, University of Nantes, 44200 Nantes, France2Department of Parasitology and Medical Mycology, IICiMed—EA1155, IRS2, University of Nantes, 44200 Nantes, France3Sorbonne Universities, UPMC Paris 06, CNRS USR3151 Protein Phosphorylation and Human Diseases, Plateforme de criblage KISSf (Kinase Inhibitor Specialized Screening facility), Station Biologique, 29688 Roscoff, France*Correspondence: pascal.marchand@univ-nantes.fr

(–)-Cercosporamide is a natural product isolated from the phytopathogen fungus *Cercosporidium henningsii* (Sugawara, F., et al. *J. Org. Chem.* **1991**, *56*, 909–910). It was identified as a broad-spectrum antifungal agent displaying an in vitro mean MIC value of 89 µg/mL (Conover, M.A., et al., *Phytochemistry* **1992**, *31*, 2999–3001) and of 10 µg/mL (Sussman, A., et al., *Eukaryot. Cell* **2004**, *3*, 932–943) against *C. albicans.* Interestingly, it appeared to act as a potent *Ca*Pkc1 ATP-competitive inhibitor with an IC_50_ of 44 nM (Sussman, A., et al., *Eukaryot. Cell* **2004**, *3*, 932–943). Furthermore, cercosporamide inhibited human PKCα (IC_50_ = 1 µM) and PKCβ (IC_50_ = 0.3 µM) (Konicek, B.W., et al., *Cancer Res.* **2011**, *71*, 1849–1857) and was later shown to inhibit other human kinases, including Mnk1/2, Jak3, GSK3β, ALK4 and Pim1, from nanomolar to low micromolar ranges (Konicek, B.W., et al., *Cancer Res.* **2011**, *71*, 1849–1857; Hou, J., et al., *Oncotarget* **2012**, *3*, 118–131).

Polyoxygenated dibenzofurans are important heteroaromatic compounds which display a wide variety of biological activities (Love, B.E. *Eur. J. Med. Chem.* **2015**, *97*, 377–387). Consequently, in continuation of our successful attempts in the search of biologically active cercosporamide inspired derivatives (Bazin, M.-A., et al. *Eur. J. Med. Chem.* **2013**, *69*, 823–832), we report here the synthesis and biological evaluation of dibenzo[*b*,*d*]furans targeting protein kinases. In addition, cercosporamide was found to recognize ATP-binding site of Mnk2 kinase through hydrogen bond network due to the 3-OH and the 4-CONH_2_ of the phenyl portion, justifying the strategy of keeping dihydroxybenzofuran-carboxamide part of the natural product model for the design of new tricyclic compounds (Hou, J., et al. *Oncotarget* **2012**, *3*, 118–131).


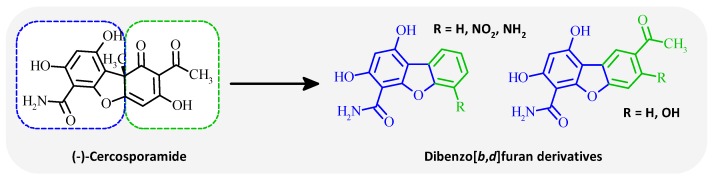


### 5.8. Fluorinated Indazole Derivatives: Promising Structures for 5-HT_4_ Receptors PET Radiotracers (FC10)

MangeantReynald^1^*StiebingSilvia^1^CaillyThomas^1^,^2^DavisAudrey^1^FosseyChristine^1^FabisFrédéric^1^CollotValérie^1^1Centre d’Etudes et de Recherche sur le Médicament de Normandie (CERMN), Normandie Univ, UNICAEN, 14000 Caen, France2Department of Nuclear Medicine, CHU Côte de Nacre, 14000 Caen, France*Correspondence: reynald.mangeant@unicaen.fr

Since its discovery in 1988, the serotonin 4 receptor subtype (5-HT_4_R) has emerged as a promising target for drug discovery and development resulting from their implications in cognition, learning, memory processes and many neuropsychiatric disorders such as Alzheimer’s disease, anxiety, depression or anorexia nervosa (Bockaert, J., et al. *A. Neuropharmacology* **2008**, *55*, 922–931.). Thus, discovery of active 5-HT_4_R agonists and antagonists remains a continuing interest in clinical research. To this end, positron emission tomography (PET) (Marner, L., et al. *Neuroimage* **2010**, *50*, 855–861.) (Caillé, F., et al. *Bioorg. Med. Chem. Lett.* **2013**, 6243–6247.) coupled with effective radioligands constitutes a valuable tool, both in clinical studies and drug discovery’s program. Based on previous works in CERMN (Lam, B.V., et al. *Chem. Eur. J*. **2016**, *22*, 4440–4446.), we aimed to develop new fluorinated indazole derivatives as potential brain 5-HT_4_R PET tracers.


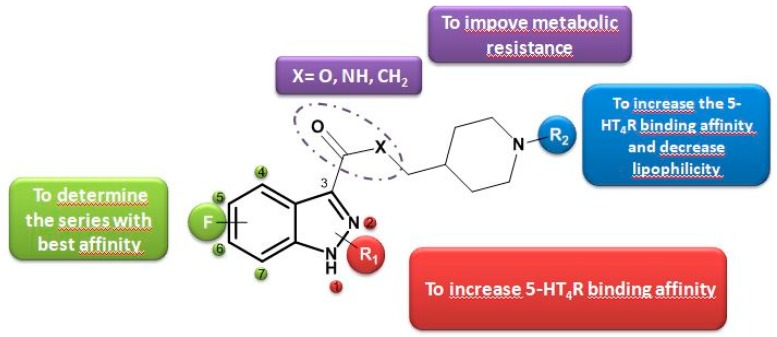


A convergent synthesis pathway to obtain fluorinated analogues has been established. A methodology allowing selective functionalization at position 3 leading to polyfunctional indazoles within a minimum of step has been developed. New pharmacomodulations studies were realized in order to increase receptor affinity, decrease lipophilicity and increase metabolic resistance.

### 5.9. Conception and Synthesis of New Pleiotropic Compounds that Both Display 5-HT_4_R Agonist Activity and Antioxidant Property in Alzheimer’s Disease (FC12)

LanthierCaroline*LecouteyCédricLalutJulienToubletFrançois-XavierDallemagnePatrickRochaisChristopheCentre d’Etudes et de Recherche sur le Médicament de Normandie (CERMN), Normandie Univ, UNICAEN, 14000 Caen, France*Correspondence: caroline.lanthier@unicaen.fr

In a world where life expectancy is increasing, Alzheimer disease (AD) is the main cause of dementia, and touch approximatively 17% of people who are more than 75 years in France. This is a progressive neurodegenerative disorder characterized by memory loss and cognitive decline. Despite the fact that the physiopathology of AD is not entirely known at the time, some molecular causes were found such as the β-amyloid peptides aggregation, tau-dependent neurofibrillary tangles, as well as oxidative stress. Currently, treatments available for patients are mainly Acetylcholine esterase (AChE) inhibitor, which only have symptomatic benefits and do not cure AD. Then there is still a strong medical need in the AD population.

In this context, the concept of Multi-Target Directed Ligands (MTDLs) was applied to design a drug with several therapeutic targets to treat a disease. The MTDL should be able in first hand, to limit the development of β-amyloid plaques obtained by the aggregation of β-amyloïd peptides (Aß). Our compounds should be able to promote the cleavage of amyloid protein precursor (APP) by a—secretase in order to produce a neuroprotective and soluble peptide sAPPa. This is the role of the 5HT_4_R agonist (blue part—fig) which is already studied in the CERMN in other MTDL projects and led to the discovery of Donecopride (Rochais, C. et al., *J. Med. Chem.* **2015**, *58*, 3172–3187). In another hand, it appears that the oxidative stress has a central role in AD (Rosini, M. et al., *J. Med. Chem.* **2014**, *57*, 2821–2831). Adding antioxidant moiety such as polyphenol or ubiquinone (orange part—fig) could trap free radicals or reactive oxygen species (ROS). To that end, different compounds will be designed and synthetized in order to evaluate their in vitro/in vivo properties regarding their agonist activity on 5-HT_4_R and antioxidant property.


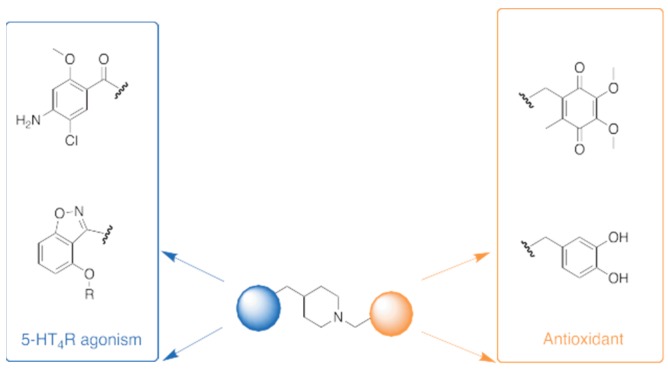


### 5.10. Antiplasmodial Activity of Polyphenolic Derivatives (FC13)

DegotteGilles^1^,^2^*AlsonSylvain G.^1^,^3^,^4^HansAurore^2^JansenOlivia^1^CieckiewiczEwa^1^RakotoarimananaHajatiana^3^RafatroHenintsoa^3^RandimbivololonaFanantenanirainy^4^FrancottePierre^2^FrederichMichel^1^1Laboratoire de Pharmacognosie, Centre Interdisciplinaire de Recherches sur le Médicament (CIRM), ULiège, Liège, Belgium2Laboratoire de Chimie Pharmaceutique, Centre Interdisciplinaire de Recherches sur le Médicament (CIRM), ULiège, Liège, Belgium3Laboratoire d’Evaluation Pharmaco-Clinique (LEPC), Institut Malgaches de Recherches Appliquées (IMRA) BP, Antananarivo, Madagascar4Laboratoire de Pharmacologie Générale, de Pharmacocinétique et de Cosmétologie (LPGPC), Faculté des sciences, Université d’Antananarivo BP, Antananarivo, Madagascar*Correspondence: gdegotte@doct.uliege.be

Despite the progress in the struggle against malaria, this parasitic disease remains a major public health problem with 216 million cases in 2016. The last advance was the development of artemisinin and its derivatives, near 15 years ago. These compounds are employed with success in combination with other antimalarial drugs and are now the recommended treatment by the *World Health Organization*. However, resistance of *Plasmodium* to these molecules appears and spreads over in Asia and Africa. Thus, the design of new antiplasmodial derivatives is imperative to hope the eradication of this infection (World Health Organisation, **2017**).

Polyphenolics compounds are well known to have multiple pharmacological activities such as antioxidant (Wright, J. S. et al., *J. Am. Chem. Soc.* **2001**, *123*, 1173–1183), antitumor (Okuda, T. in Food Factors for Cancer Prevention **1997**, 280–285), antimicrobial (Bisignano, G. et al., *J. Pharm. Pharmacol.* **1999**, *51*, 971–974) and antiplasmodial (Köhler, I. et al., *Z. Naturforsch*. **2002**, *57c*, 277–281.). Regarding this last effect, the screening of caffeic acid (**1**) and its derivatives was performed in vitro and in vivo. These evaluations permitted to select ethyl caffeate (**2**) as the most potent derivatives against a 3D7 strain in culture. More interestingly, this ester was found active in mice with a stage specificity on the young trophozoites. The major interest of this molecule is that caffeic acid is widely distributed in plants and is considered non-toxic.


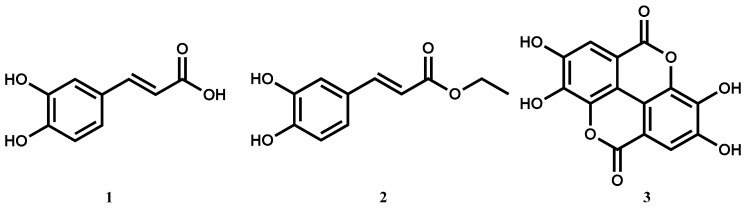


Structures of caffeic acid (1), ethyl caffeate (2) and ellagic acid (3).

Considering the results obtained with this first series of polyphenolic analogs and based on the known antimalarial activity of ellagic acid (**3**) in vitro and in vivo (Soh, P. N. et al., *Antimicrob. Agents Chemother*. **2009**, *53*, 1100–1106.), we currently investigate this widely distributed polyphenol as a scaffold for further pharmacomodulation. The new structures will be screened to determine their antiplasmodial effect.

**Acknowledgments:** This work was financed by FNRS.

### 5.11. Chemical Modifications of Mycobacterium Tuberculosis MabA Inhibitors (FC16)

FaïonLéo^1^*PintialaCatalin^1^MouneMartin^2^FritaRosangela^2^DjaoutKamel^2^PiveteauCatherine^1^DeprezBenoit^1^BaulardAlain^2^WillandNicolas^1^FlipoMarion^1^1Univ. Lille, Inserm, Institut Pasteur de Lille, U1177—Drugs and Molecules for living Systems, F-59000 Lille, France2Univ. Lille, CNRS, Inserm, CHU Lille, Institut Pasteur de Lille, U1019—UMR 8204—CIIL—Center for Infection and Immunity of Lille, F-59000 Lille, France*Correspondence: leo.faion@univ-lille2.fr

This infection, caused by *Mycobacterium tuberculosis*, kills each year 1.7 million people (World Health Organization, Global Tuberculosis Report, 2017, http://www.who.int/tb/publications). A treatment, involving a combination of drugs for a minimum of six months, is available but the rapid emergence of multidrug resistant (MDR) strains of *M. tuberculosis* stresses the need for alternative therapies. The enzyme MabA (FabG1) is involved in the biosynthesis of mycolic acids (Marrakchi, H, et al. *A. Microbiology* **2002**, *148*, 951), which are very long-chain fatty acids playing a major role in the bacterial cell wall. The gene coding for this enzyme has been shown to be essential for M. tuberculosis (Parish, T. *J. Bacteriol.* **2007**, *189*, 3721), therefore our goal is to find drug-like inhibitors of MabA that prevent mycobacterial growth. The screening of a 1280 fragment-library on MabA using a new enzymatic assay allowed the identification of several families of inhibitors. One of this series was selected for further optimization and structure-activity relationships were established. This led to the discovery of the first low-molecular-weight MabA inhibitors. The synthesis of these compounds and the biological results will be presented.


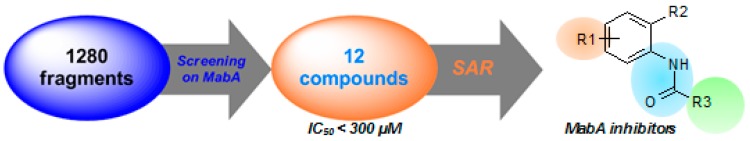


### 5.12. Mcl-1 Selective Inhibitor Modulation: Discovery of Mcl-1/Bcl-xL Dual Inhibitors, for the Treatment of Chemoresistant Ovarian Cancers (FC17)

De PascaleMartina^1^*DenisCamille^1^BrotinEmilie^2^PoulainLaurent^2^Voisin-ChiretAnne-Sophie^1^1Centre d’Etudes et de Recherche sur le Médicament de Normandie (CERMN), Normandie Univ, UNICAEN, 1400 Caen, France2Plate-forme ImpedanCELL, Inserm U1086 ANTICIPE, axe BIOTICLA, Centre de Lutte Contre le Cancer François Baclesse, 14000 Caen, France*Correspondence: martina.depascale@unicaen.fr

Protein-protein interactions (PPIs) are attractive targets because they control numerous cellular processes. In particular, in oncology, our laboratory is interested in Bcl-2 family proteins, apoptosis regulators. This family is composed by pro-apoptotic (Bax, Bak, Noxa…) and anti-apoptotic members (Bcl-2, Bcl-x_L_, Mcl-1…) that interact via PPIs to regulate the cellular death thanks to BH domains (BH1, BH2, BH3). In solid human tumors, the anti-apoptotic members are over expressed and their inhibition represents a novel and promising strategy for new anticancer drugs. In ovarian carcinoma, it has been previously demonstrated that Bcl-x_L_ and Mcl-1 cooperate to protect tumor cells against apoptosis and their concomitant inhibition leads to massive apoptosis even in absence of chemotherapy. In this context, we have designed and synthesized an oligopyridine BH3-mimetic, named pyridoclax (Gloaguen, C., et al. *J. Med. Chem.* **2015**, *58*, 1644–1668) that interacts selectively with Mcl-1 hydrophobic pocket.^1^ Know that Bcl-x_L_ inhibition is also accessible using BH3-mimetics, we would design a novel class of BH3-mimetics able to inhibit simultaneously Bcl-x_L_ and Mcl-1 (Zhang, Z., et al. *Arch. Pharm. Chem. Life Sci.* **2015**, *348*, 1–11). We will present our approach to design such dual inhibitors from pyridoclax scaffold taking into account the structure-activity relationship.


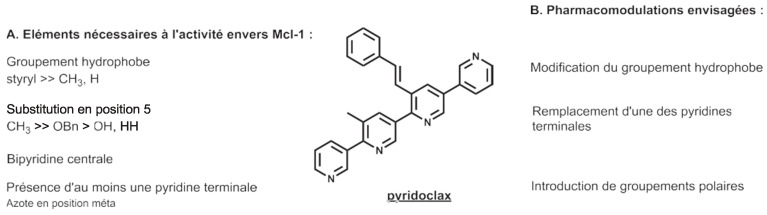


### 5.13. Benzoylthiosemicarbazides as Promising Antimicrobial Agents Targeting D-alanine-D- alanine Ligase in bacterio (F18)

AmeryckxAlice^1^*ThabaultLéopold^1^PochetLionel^2^Van BambekeFrançoise^1^FrédérickRaphael^1^1Medicinal Chemistry Research Group/Cellular and Molecular Pharmacology, Louvain Drug Research Institute (LDRI), Université catholique de Louvain, Av. E Mounier 74, 1200 Woluwé-Saint-Lambert, Belgium2Department of Pharmacy, Namur Medicine & Drug Innovation Center (NAMEDIC), Namur Research Institute forLife Sciences (NARILIS), University of Namur, Namur, Belgium*Correspondence: alice.ameryckx@uclouvain.be

As the phenomenon of antibiotic resistance is dramatically increasing these days, searching for new therapeutic targets less vulnerable to this resistance issue appears as a real need (Cassell, G. H., et al., *JAMA* **2001**, *285*, 601–605). The cell wall of bacteria and the enzymes that are involved in its synthesis are promising targets for many antibiotics, which inhibit the late stages of peptidoglycan biosynthetic pathway (Fan, C., et al., *Science* **1994**, *266*, 439–443). But the resistance phenomena have revealed the high flexibility in this assembly pathway, and the need to target other enzymes acting on earlier steps of peptidoglycan synthesis (Tytgat, I., et al., *Curr. Med. Chem.* **2009**, *16*, 2566–2580) D-alanyl-D-alanine ligase (Ddl) is of particular interest as it utilizes a substrate (D-alanine) which is specific for bacterial peptidoglycan biosynthesis and essential for bacterial growth (Zawadzke, L. E., et al., *Biochemistry* **1991**, *30*, 1673–1682; Gholizadeh, Y., et al., *Protein Sci.* **2001**, *10*, 836–844).

In this work, a series of 37 benzoylthiosemicarbazides inhibitors of D-Ala-D-Ala ligase (Ddl) were designed and synthesized in order to target resistant strains of bacteria. Among these, the 4-(3,4-dichlorophenyl)-1-(2-hydroxybenzoyl)-3-thiosemicarbazide (**1**) was identified as a very potent Ddl inhibitor with an activity in the micromolar range. This compound, possessing strong antimicrobial activities including against multidrug resistant strains, was proved to act through a bactericidal mechanism and demonstrated very low cytotoxicity on THP-1 human monocytic cell line.


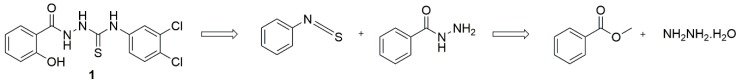


Further structure-activity relationships (SARs) studies provided evidence that the hydroxyl substituent in the 2-position (R_1_) of the benzoylthiosemicarbazide scaffold is essential for the enzymatic inhibition. The in vivo biochemical mechanism was then determined by UPLC-MS dosage (S. Putty, A., et al., *Chem. Biol. Drug. Des.* **2011**, *78*, 757–763) of intracellular L-Ala, D-Ala and D-Ala-D-Ala levels in response to 4-(3,4-dichlorophenyl)-1-(2-hydroxybenzoyl)-3- thiosemicarbazide (**1**). Finally, these encouraging results prompted us to investigate the selectivity of this compound towards various enzymes and receptors. This work thus highlights the thiosemicarbazide motif as very promising for the development of novel antibacterial compounds acting through an interesting mechanism of action, good selectivity and low cytotoxicity.

## 6. Posters

### 6.1. PfRab6 and PfPyrK1: New Putative Plasmodial Targets for Designing Novel Antimalarials (P01)

AmraneDyhia^1^PrimasNicolas^1^*LanzadaGilles^1^HutterSébastien^2^AzasNadine^2^VerhaeghePierre^3^VanellePatrice^1^1Aix-Marseille Univ, CNRS, ICR UMR 7273, Pharmaco-Chimie Radicalaire, Faculté de Pharmacie, 13385 Marseille, France2Aix-Marseille Univ, IHU Méditerranée Infection, UMR VITROME, 13005 Marseille, France3Université Paul Sabatier, CNRS UPR 8241, Laboratoire de Chimie de Coordination, 31077 Toulouse, France*Correspondence: nicolas.primas@univ-amu.fr

Malaria is still responsible for a major burden on human health and is becoming increasingly difficult to treat due to the emergence of drug resistance toward Artemisinin-based Combination Therapy (WHO, World Malaria Report, **2017**). In order to discover new antimalarials with an original mechanism of action, our laboratory previously described the synthesis and the biological activities of a 2-trichloromethylquinazoline hit active against the multiresistant K1 *P. falciparum* strain (Verhaeghe, P., et al. *Bioorg. Med. Chem.* **2009**, *17*, 4313–4322). A chemoproteomics approach was used in order to elucidate its mechanism of action as it was different from the ones of commercial antimalarials. Two putative plasmodial targets were identified—PfRab6 and PfPyrK1—which are essential for the parasite. The confirmation of the mechanism of action implicating these two proteins is in progress and pharmacomodulation refinement is still required.


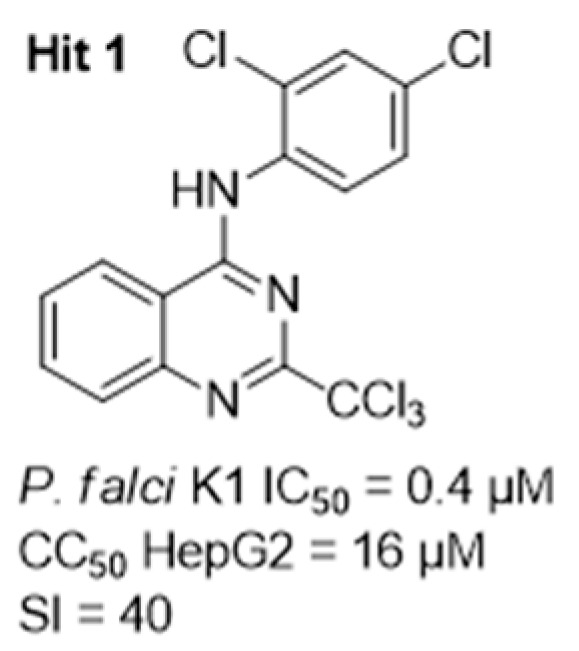


In previous work, we report that the 4-sulfonamidoquinazoline derivatives were unactive (Primas, N., et al., *Molecules* **2012**, *17*, 8105–8117). Isosteric replacement with a carboxamide substituent showed promising activity. Thus, we prepared a new library of aliphatic, aryl or heteroaryl carboxamide derivatives. Moreover, a scaffold hopping strategy previously showed that the replacement of the quinazoline moiety by a quinoxaline one improved the cytotoxicity profile (Desroches, J., et al., *Eur J. Med. Chem.* **2017**, *125*, 68–86). Thus, we synthetized a new series of 2-trichloromethylquinoxalines bearing either arylether, arylthioether or aniline substituents at position 3. We report herein this new pharmacomodulation work: the synthesis of the corresponding derivatives and the biological results will be detailed in the poster.


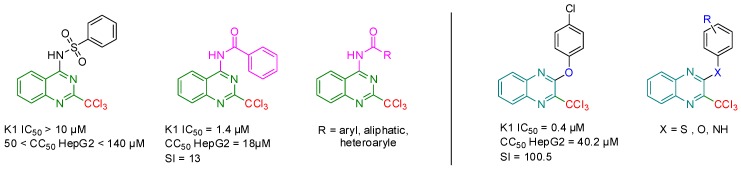


**Acknowledgments:** This work was supported by the ANR NINTARMAL project, grant ANR-17-CE11-0017 of the French Agence Nationale de la Recherche.

### 6.2. Synthesis and Antiparasitic Evaluation of New 2,9-bis[(substituted-aminomethyl)phenyl]-1,10-phenanthroline Derivatives (P05)

GuillonJean^1^*CohenAnita^2^DasRabindra Nath^1^BoudotClotilde^3^MoreauStéphane^1^RongaLuisa^1^SavrimoutouSolène^1^ElmiLilian^1^RubioSandra^1^Dassonville-KlimptAlexandra^4^AzasNadine^2^CourtiouxBertrand^3^MergnyJean-Louis^1^MulliéCatherine^4^SonnetPascal^4^1Université de Bordeaux, INSERM U1212, UMR CNRS 5320, ARNA Laboratory, UFR des Sciences Pharmaceutiques, Bordeaux, France2Aix-Marseille Univ, IRD, AP-HM, SSA, VITROME, Marseille, France3INSERM U1094, Tropical Neuroepidemiology, Université de Limoges, Institute of Neuroepidemiology and Tropical Neurology, Limoges, France4Université de Picardie Jules Verne, UFR de Pharmacie, AGIR—EA 4294 Amiens, France*Correspondence: jean.guillon@u-bordeaux.fr

Malaria represents one of the most prevalent parasitic infections worldwide. In 2017, an estimated 219 million cases of malaria occurred all over the world and there were an estimated 435,000 deaths from malaria globally (World Health Organization, World Malaria Report **2018**). The increase in drug resistance in the malaria endemic areas has significantly reduced the potency of most currently used antimalarial compounds. Thus, new antimalarial drugs with new potential mechanisms of action are now required to overcome this emerging resistance and also to control an ever-increasing number of epidemics due to the parasites (Biot, C. et al., *Infect. Disorders-Drug Targets* **2006**, *6*, 173–204; Barnett, D.S., et al., *Chem. Rev.* **2014**, *114*, 11221–11241). Following this strategy, a series of new 2,9-bis[(substituted-aminomethyl)phenyl]-1,10-phenanthroline derivatives **1a-s** was synthesized, and these corresponding derivatives were biologically evaluated in vitro against three protozoan parasites (*Plasmodium falciparum*, *Leishmania donovani* and *Trypanosoma brucei brucei*). Obtained results showed antiparasitic activity with IC_50_ values in the μM range. In order to assess the effective selectivity of this series, the in vitro cytotoxicity of these molecules was also evaluated against human HepG2 cells; for some derivatives, cytotoxicity was observed at significantly higher concentrations than antiparasitic activity, revealing an interesting selectivity index (Guillon, J. et al., *Chem. Biol. Drug Des.* **2018**, *91*, 974–995).


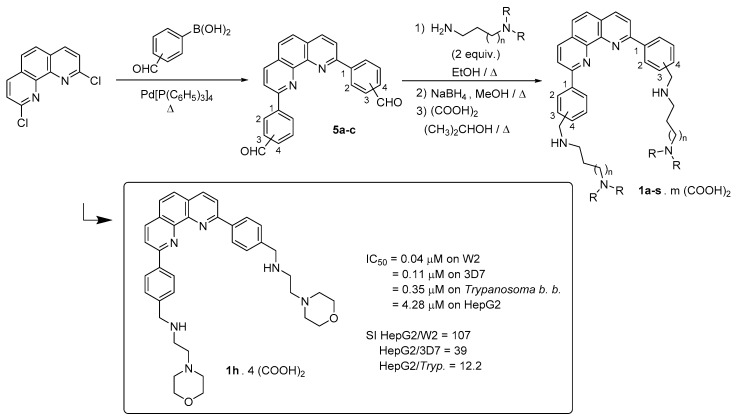


The 2,9-bis[(substituted-aminomethyl)phenyl]-1,10-phenanthroline **1h** was identified as the most potent antimalarial candidate, with cytotoxic to antiparasitic activity ratios of 107 and 39 against a chloroquine-sensitive and a chloroquine-resistant strain of *P. falciparum*, respectively. As the telomeres of the parasite *P. falciparum* are the likely target of this compound, we also investigated stabilization of the Plasmodium telomeric G-quadruplexes by our phenanthroline derivatives through a FRET melting assay. Consequently, the ligands **1f** and **1m** were noticed to be more specific for FPf8T with higher stabilization for FPf8T than for the human F21T sequence.

**Acknowledgments:** We thank the DGA and ANR (projects ANR-12-ASTR-003, OligoSwitch [ANR-12-IS07-0001], “Quarpdiems” [ANR-12-BSV8-0008-01], and “VIBBnano” [ANR-10-NANO-04-03]) for financial support of this study.

### 6.3. Development of a Binding Assay Using Fluorescence Polarization (P06)

JouanneMarie^1^*DenisCamille^1^De PascaleMartina^1^KiefferCharline^1^WeiswaldLouis-Bastien^2^Sopková-de Oliveira SantosJana^1^PoulainLaurent^2^Voisin-ChiretAnne Sophie^1^1Centre d’Etudes et de Recherche sur le Médicament de Normandie Normandie Univ, UNICAEN, CERMN, 14000 Caen, France2Inserm U1086 « ANTICIPE » Unité de Recherche Interdisciplinaire pour la Prévention et le Traitement des Cancers. Axe 2: Biologie et Thérapies Innovantes des Cancers Localement Agressifs (BioTICLA), Université de Caen Normandie, UFR Santé et SF 4206 ICORE, 14000 Caen, France*Correspondence: marie.jouanne@unicaen.fr

For few years, our laboratory is interested in design and synthesis of small molecules as potential modulators of Protein Protein Interactions (PPIs). Particularly, we are interested in proteins involved in apoptosis process such as anti-apoptotic Bcl-2 family proteins or more recently the X-linked inhibitor of apoptosis protein (XIAP).

Based on its expertise in molecular modelling and in medicinal chemistry, our team has developed a large library of abiotic foldamers able to mimic the secondary structures of proteins (Voisin-Chiret, A.S., et al., *Tetrahedron* **2012**, *68*, 4381–4389; Voisin-Chiret, A.S., et al., *Pure Appl. Chem.* **2012**, *84*, 2467–2478; Sopkova-de Oliveira Santos, J., et al., *J. Chem. Inf. Model.* **2012**, *52*, 429–439; Jouanne, M., et al., *Eur J. Org. Chem.* **2016**, *34*, 5686–5696). The biological evaluation of these compounds has been realized with our partner (binding affinity by SPR, cellular tests…) and some of them have been shown very interesting anti-proliferative activities against Mcl-1 protein (Gloaguen, C., et al., *J. Med. Chem.* **2015**, *58*, 1644–1668). New molecules are still synthesized in order to disturb targeted proteins. In front of the amount of synthesized compounds, we decided recently to implement screening binding affinities by fluorescence polarization to obtain a first result to select best compounds before to perform cellular and in vivo tests and to improve structure-activity relationship.


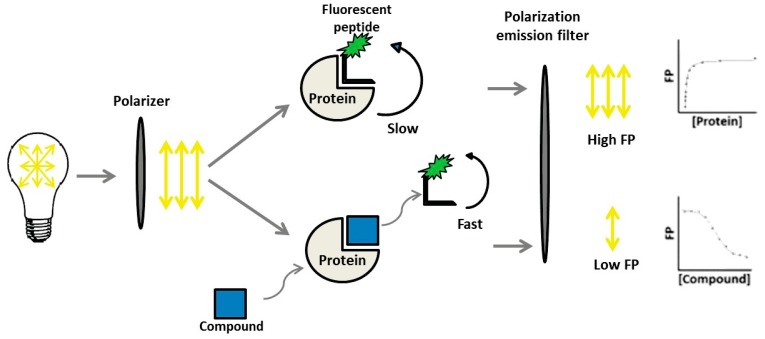


**Acknowledgments:** The authors are grateful for financial support provided by Regional council of Normandy, FEDER, the University of Caen and the Ministry of Research.

### 6.4. C-H (Radio-)iodination of N-acylsulfonamides, Access to New Iodinated Radiotracers in Cancerology (P07)

HebertAlexandra^1^*BenoistFlorian^1^DubostEmmanuelle^1^FabisFrédéric^1^CaillyThomas^1^,^2^1Centre d’Etudes et de Recherche sur le Médicament de Normandie CERMN UNICAEN EA 4258 FR CNRS 3038 INC3M SF 4206 ICORE Boulevard Becquerel, University of Caen Normandie, 14032 Caen, France2Department of Nuclear Medicine, CHU Cote de Nacre, 14000 Caen, France*Correspondence: alexandra.hebert@unicaen.fr

Labelling of (bio)molecules with radioactive isotopes is of high interest to the scientific community, as it strongly impacts the discovery process in life science and nuclear medicine. Radiolabelled molecules have been extensively used to assess biochemical reactions, to measure in vivo distribution of a substance or to perform RadioImmunoAssay (RIA). In nuclear medicine, radio-therapeutics for RadioIsotope Therapy (RIT) and radio-tracers for molecular imaging experiments such as Positron Emission Tomography (PET), Single Photon Emission Computed Tomography (SPECT) or scintigraphy have been described. Several useful isotopes of iodine can be used for both diagnosis and therapy: ^123^I for SPECT imaging, ^124^I for PET imaging, ^125^I for biological assays and nuclear medicine and ^131^I for radio-therapy and scintigraphy. Our group has recently developed a method to radio-iodinate N-acylsulfonamides through a room temperature palladium mediated C-H radio-iodination. This original strategy allows radiolabelling in very mild conditions without the use of chemical precursors.


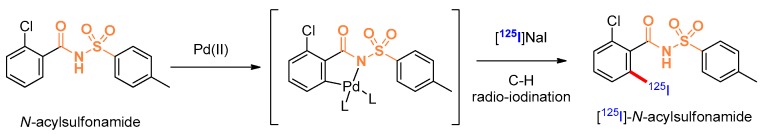


In this context, we are currently enlarging the scope of this methodology toward the radio-iodination of antitumoral agents containing a *N*-acylsulfonamide group. Thus, an antiproliferative agent LY32262 (Nathubhai, A. et al., *J. Med. Chem*. **2016**, *60*, 814–820), a Bcl-x_L_/Mcl1 dual inhibitor (Cominetti, M.D.D. et al., *Org. Biomol. Chem.* **2016**, *14*, 10161–10164) and ABT-737 (Broughton, L.J. et al. *J. Photochem. Photobiol. B* **2016**, *163*, 374–384) have been selected and progress toward the (radio-)iodination of these molecules will be presented.


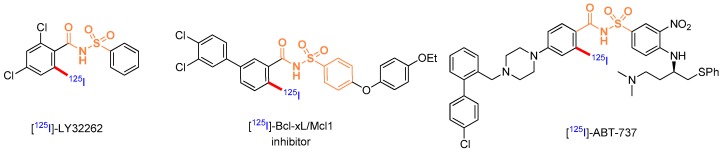


### 6.5. New Multifunctional Diamine Drugs to Prevent Oxidative and Carbonyl Stress Extension in Alzheimer’s Disease (P08)

LohouElodie^1^*SasakiN. André^1^BoullierAgnès^2^,^3^,^4^SonnetPascal^1^1Laboratoire AGIR, UFR de Pharmacie, Université de Picardie Jules Verne, F-80037 Amiens, France2UFR de Médecine, Université de Picardie Jules Verne, F-80037 Amiens, France3MP3CV, Centre Universitaire de Recherche en Santé (CURS), F-80054 Amiens (Salouël), France4CHU Amiens Picardie, F-80054 Amiens (Salouël), France*Correspondence: elodie.lohou@u-picardie.fr

Carbonyl stress is now considered to play an important role in Alzheimer’s disease (AD) pathogenesis. Indeed, extensive Advanced Glycation Endproducts (AGEs) and Advanced Lipid peroxidation Endproducts (ALEs) accumulation linked to enhanced reactive carbonyl species (RCS) level has been reported in amyloid *β* (A*β*) plaques and tau-associated neurofibrillary tangles (NFT). In AD, A*β*-oligomers induce oxidative stress whereas transition metals (Zn^2+^, Cu^2+^ and Fe^3+^) stimulate A*β* aggregation and amyloid precursor protein (APP) processing. RCS such as methylglyoxal (MGO) or malondialdehyde (MDA) are endogenously formed during the sugar glycoxidation and lipid peroxidation of polyunsaturated fatty acids induced by oxidative stress exacerbation. Their condensation with amino groups of tissue proteins gives AGE and ALE. Consequently, carbonyl stress takes part in the vicious downward redox amyloid spiral leading to neurodegeneration as toxic mediator and oxidative stress promoter (Butterfield, D.A., et al. *Trends Mol. Med.* **2001**, *7*, 548–554; Tiiman, A., et al., *Neurochem. Int.* **2013**, *62*, 367–378). Glycated A*β* cross-linking promotion accelerates its deposition and its protease resistance. Moreover, AGE promote via their receptors RAGE oxidative stress and inflammation as well as cell apoptosis (Krautwald, M. et al., *Exp. Gerontol.* **2010**, *45*, 744–751).

Considering the multifactorial pathogenesis of AD, we designed new multifunctional diamine drugs that are simultaneously able to trap RCS (primary vicinal diamine function) as well as ROS and biometals (phenolic acid or hydroxypyridinone (HOPO) moiety) (Lohou, E. et al., *Eur. J. Med. Chem.* **2016**, *122*, 702–722). In the poster, synthesis of these new promising hybrid AGE/ALE inhibitors, but also their physicochemical and biological evaluation in terms of their carbonyl trapping capacity, antioxidant activity, Cu^2+^-chelating capacity as well as their protective properties against in vitro MGO-induced apoptosis in the model AD cell-line PC12 are reported.


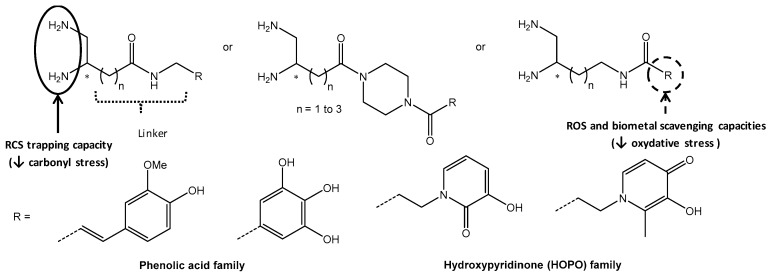


**Acknowledgments:** We thank “SATT Nord” for financial support of this study.

### 6.6. From the Design to the In Vitro Evaluation of New Iodinated Ligands as Potential 5-HT_4_ SPECT Radiotracers (P09)

BabinVictor^1^*TournierBenjamin^2^DavisAudrey^1^MilletPhilippe^2^CaillyThomas^1^BouillonJean-Philippe^3^FabisFrédéric^1^1Normandie University, UNICAEN, CERMN, 14000 Caen, France2Hôpitaux Universitaires de Genève, Département de Santé Mentale et de Psychiatrie, Service de Psychiatrie Générale, Unité des Biomarqueurs de Vulnérabilité, Chemin du Petit-Bel-Air, 2 CH-1225 Genève, Suisse3Normandie Univ, UNIROUEN, INSA Rouen, CNRS, COBRA UMR 6014, F-76000 Rouen, France*Correspondence: victor.babin@unicaen.fr

Serotonin (5-hydroxytryptamine 5-HT) is a neurotransmitter acting on the central and peripheral nervous system through a large variety of receptors subdivided in seven families. The serotonin receptor subtype-4 (5-HT_4_R) is known to be involved in physiological and pathological disorders such as Alzheimer disease (Lezoualc’h, F., *Exp. Neurol.* **2007**, *205*, 325–329). The development of radiotracer is essential for the evaluation of new drugs targeting 5-HT_4_R and also for investigation of the receptor involvement in a variety neurodegenerative and neuropsychiatric disorders.

Since a few years, our group has been involved in the development of new Single Photon Emission Computed Tomography (SPECT) radiotracers targeting the 5-HT_4_R and many iodinated ligands based on phenanthridine (Dubost, E., et al. *J. Med. Chem.* **2012**, *55*, 9693–9707) and diazaphenanthridine (Fresneau, N. et al. *Eur. J. Med. Chem*. **2015**, *94*, 386–396) scaffolds have been designed. An efficient synthetic access to these compounds has been established and many potent ligands have been synthetized. Biological evaluation has led to a “hit” compound and this ligand and some analogues have been labeled with 125-iodine for SPECT imaging but to date none of these radioligands have been able to image the 5-HT_4_R specifically. In order to obtain an efficient 5-HT_4_R radiotracer, our group has decided to synthesize a series of new iodinated ligands with reduced lipophilicity to promote in vivo specificity.


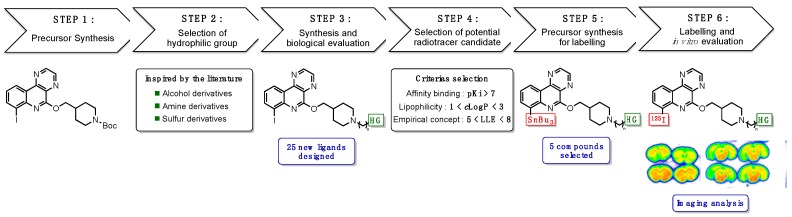


### 6.7. Fragment-Based Screening Approach Leading to the Inhibition of Matrix Metalloproteinase MT5-MMP (P10)

LalutJulien^1^*GiovanniniJohanna^1^SuzannePeggy^1^LepailleurAlban^1^BureauRonan^1^BarangerKevin^2^RiveraSantiago^2^DallemagnePatrick^1^BriereJean-François^3^RochaisChristophe^1^1Centre d’Etudes et de Recherche sur le Médicament de Normandie (CERMN), Normandie Univ, UNICAEN, 14000 Caen, France2Institute of Neuropathophysiology (INP), UMR7051, CNRS, Aix Marseille Université, Marseille, France3Normandie Univ, UNIROUEN, INSA Rouen, CNRS, COBRA UMR 6014, F-76000 Rouen, France*Correspondence: julien.lalut@unicaen.fr

Fragment-based drug design is a promising and modern concept in drug discovery which allows to obtain affine and selective ligands for many biological targets from small molecules chosen as starting points. With less complex structures, fragments reach inaccessible binding sites and have more points of interaction contrary to the larger lead candidates developed in traditional medicinal chemistry or generated from HTS. However, fragments generally have less affinity for their targets, that’s why it is necessary to grow the molecules in order to obtain the desired affinity with favorable selectivity and ADME properties (Wermuth, C.G., et al., *The Practice of Medicinal Chemistry*, *4th Edition*, Academic Press: London, UK **2015**).


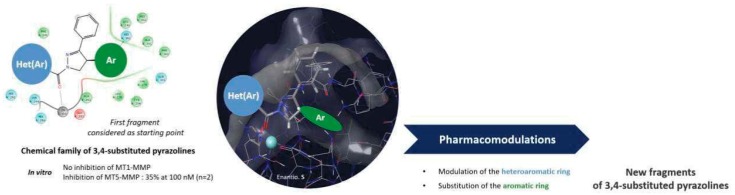


While the majority of screened chemical libraries are essentially linear or planar fragments, we have decided to focus our attention on the screening of three-dimensional geometry molecules from different research programs with scaffolds able to distribute substituents spatially (Mahé, O., et al., *Ang. Chem. Int. Ed.* **2010**, *49*, 7072–7075; Gembus, V., et al., *Org. Biomol. Chem.* **2010**, *8*, 3287–3293). Several common techniques (NMR, cristallography…) can be used for the identification of a hit from a library of fragments. Our original chemical library has been screened in silico and in vitro to identify fragments interacting towards MT5-MMP, a matrix metalloprotease recently identified as a potential relevant target for the treatment of Alzheimer’s disease (Baranger, K., et al., *Cell. Mol. Life Sci.* **2016**, *73*, 217–236).

A 3,4-substituted pyrazoline derivative was identified from screenings as a first hit, especially with a selectivity towards MT5 vs. MT1. Molecular docking seems to indicate a better score for the *S* enantiomer. Their separation as well as other pharmacomodulations are underway to increase the affinity of this first fragment.

### 6.8. Anti-Trypanosomatid Lead Optimization in 3-nitroimidazo[1,2-a]pyridine series (P11)

FersingCyril^1^BoudotClotilde^2^PedronJulien^3^HutterSébastien^4^Paoli-LombardoRomain^1^PrimasNicolas^1^*Castera-DucrosCaroline^1^Bourgeade-DelmasSandra^5^Sournia-SaquetAlix^3^WyllieSusan^6^FairlambAlan^6^ValentinAlexis^5^RathelotPascal^1^AzasNadine^2^CourtiouxBertrand^4^VerhaeghePierre^3^VanellePatrice^1^1Aix-Marseille Univ, CNRS, ICR UMR 7273, Pharmaco-Chimie Radicalaire, Faculté de Pharmacie, 13385 Marseille, France2Université de Limoges, UMR Inserm 1094, Neuroépidémiologie Tropicale, 87025 Limoges, France3Université Paul Sabatier, CNRS UPR 8241, Laboratoire de Chimie de Coordination, 31077 Toulouse, France4Aix-Marseille Univ, IHU Méditerranée Infection, UMR VITROME, 13005 Marseille, France5Université Paul Sabatier, IRD UMR 152, PHARMA-DEV, 31400 Toulouse, France6Division of Biological Chemistry and Drug Discovery, Wellcome Trust Building, School of Life Sciences, University of Dundee, Dundee, Scotland, UK*Correspondence: nicolas.primas@univ-amu.fr

Among the drugs currently developed to treat parasitic diseases caused by trypanosomatids such as *Leishmania* and *Trypanosoma*, the nitrodrug fexinidazole exerts its mechanism of action via a reductive bioactivation step, leading to cytotoxic metabolites. This reaction is catalyzed by nitroreductases (NTR) enzymes expressed by these protozoa (Wyllie, S., et al. *Antimicrob. Agents Chemother.* **2013**, *57*, 901–906; Wyllie, S., et al., *PLoS Pathog*. **2016**, *12*, e1005971).

In order to design new NTR substrates, we synthesized original 3-nitroimidazo[1,2-*a*]pyridines and highlighted a hit molecule (Castera-Ducros, C., et al. *Bioorg. Med. Chem*. **2013**, *21*, 7155–7164). A pharmacomodulation work afforded an antileishmanial lead compound, functionalized at the C8 position by a *p*-chlorophenylsulfane substituent and displaying both a very good activity against three species of *Leishmania* and a low cytotoxicity toward the HepG2 and THP1 cell line. This molecule is selectively activated by the type 1 NTR of *Leishmania*, and shows neither mutagenic effect in the Ames test nor genotoxic properties in the comet assay. It has a poor microsomal stability, with minimal influence on the antiparasitic potential as the probable metabolites (sulfoxide and sulfone, similarly to fexinidazole) remain active. More recently, an antitrypanosomal Lead molecule bearing a 4-hydroxybutyn-1-yl substituent at C8 was discovered: also activated by a trypanosomal NTR, this compound displayed an improved microsomal stability, along with a better hydrosolubility. Therefore, this molecule showed suitability for an in vivo evaluation, currently undertaken on a *Trypanosoma brucei* infected mouse model. Further pharmacomodulation works are also conducted in the aim of optimizing both the activity and the PK properties of these anti-trypanosomatid 3-nitroimidazo[1,2-*a*]pyridines.


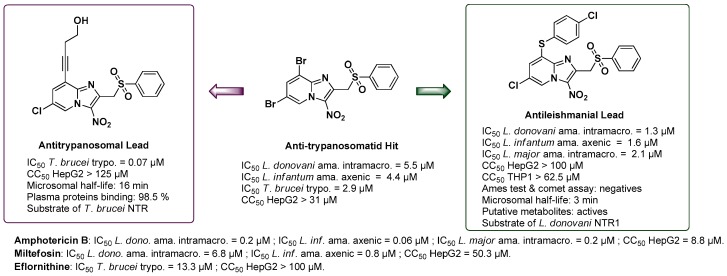


### 6.9. Development and Characterization of a Versatile Nanoemulsion of Interest for Pre-Clinical Assays (P12)

SéguyLine*GrooAnne-ClaireDemissyEtienneMalzert-FréonAurélieCentre d’Etudes et de Recherche sur le Médicament de Normandie (CERMN), Normandie Univ, UniCaen, 14000 Caen, France*Correspondence: line.seguy@unicaen.fr

About half of new chemical entities entering drug development pipelines are poorly water soluble. To overcome this serious hurdle and to limit the number of lead compounds or drug candidates to be eliminated from discovery pipelines, formulation strategies can be envisaged as soon as possible in drug discovery to enhance the solubility, and in fine the bioavailability of compounds. Among formulations to envisage, nanoemulsions refer to nanosized (typically below 300 nm) droplets of oil dispersed in water. They can be considered as a perfect delivery system for high lipophilic drugs since they permit high drug loading. In the same time, nanoemulsions assure protection of the drug, reduce its intrinsic toxicity, and even promote a prolonged drug release and an increased bioavailability. In regard of these various advantages, formulation of drug-loaded nanoemulsions appears particularly appealing from the early drug discovery to permit early animal experiments.

Nanoemulsions that we want to develop must be administrable by different routes (oral or parenteral routes), and hence must be safe, easily sterilisable, with adapted granulometric properties (monodisperse diameter of about 100 nm), with neutral surface charge, with pH and osmolarity compatible with parenteral adminstration, and transposable to many drugs with various physico-chemical properties.

In regard of these objectives, nanoemulsions were prepared with GRAS excipients by spontaneous emulsification method, a low-energy process requiring no sophisticated equipment, and without using any organic solvent. In order to define appropriate excipients, an in vitro test was set up in the laboratory to define hemolytic properties on human blood cells of excipients used alone or formulated. Among the twenty excipients tested, a medium chain triglyceride and two surfactants were selected for formulation assays. The feasibility of nanoemulsions with appropriate properties was determined by varying process parameters and the relative proportion of excipients. Based on a ternary phase diagram, the best formulation with optimal granulometric properties was defined. Encapsulation assays of a model drug were performed, and the stability of the formulation was determined in time, and also after dilution in various complex biomimetic media.

From the results, it appears that the developed nanoemulsions present appropriate properties for oral and parenteral administration routes. Thus, they would provide a valuable option as formulation strategy for drug discovery.

### 6.10. Conception of Pleiotropic (pro)Drugs MAO-B Inhibitor/AChE Inhibitor for Alzheimer’s Disease (P14)

GuieuBenjamin*LecouteyCédricToubletFrancois-XavierChristopheRochaisPatrickDallemagneCentre d’Etudes et de Recherche sur le Médicament de Normandie (CERMN), Normandie Univ, UNICAEN, 14000 Caen, France*Correspondence: benjamin.guieu@unicaen.fr

Alzheimer’s disease (AD) is a multifactorial neurodegenerative disorder, leading to the most common (Anand, R., et al. *Neuropharmacology* **2014**, *76*, 27–50) form of dementia in the elderly. Given AD’s multifactorial causes, the classical pharmacological approach consisting in interacting very selectively with a single target has shown clinical limitations, failing to restore such a complex biological system. As a result, more and more examples illustrate the concept of Multi-Target Directed Ligands (MTDLs), molecules which display several activities by interacting with different biological in order to obtain a synergy of action (Cavalli, A., et al. *J. Med. Chem.* **2008**, *51*, 347–372). For example, our group described the donecopride, first MTDL inhibiting acetylcholinesterase (AChE) and activating 5-HT_4_ serotoninergic receptors, and now in preclinical evaluation (Lecoutey, C., et al., *Proc. Natl. Acad. Sci. USA* **2014**, *111*, E3825–E3830).

Ladostigil is a novel type of MTDL currently in phase II clinical trials. It is the first compound able to release an active compound, hydroxyrasagiline, a MonoAmine Oxidase B (MAO-B) inhibitor, through the interaction with a first target, the acetylcholinesterase (AChE), which is temporarily inhibited during the process (Youdim, M. B. *Curr. Alzheimer Res.* **2006**, *3*, 541–550).

The objective of this project is to illustrate the concept the concept of molecules which are both drugs and prodrugs: the action on a first target will release a second agent acting on another target. Our objective remains original by leading to the design of (pro)drugs interacting with MAO-B to inhibit it and release another anti-AD drug. Using AChE inhibitors, we may then obtain an action similar to ladostigil but in an opposite mechanistic way.


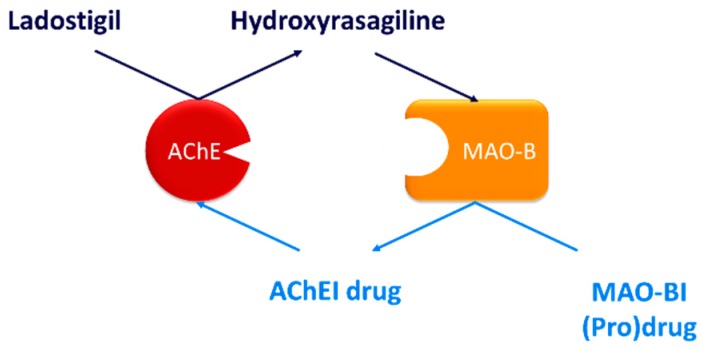


### 6.11. Enhancing the Potency of Aminoquinolinealcohols toward Gram-Negative Bacteria with Conjugated Peptides (P15)

LaumailléPierreDassonville-KlimptAlexandra*Da-NascimentoSophiePeltierFrançoisMulliéCatherineCastelainSandrineSonnetPascalAGIR (AGents antiInfectieux Résistances et chimiothérapie), Université de Picardie Jules Verne, 80037 Amiens, France*Correspondence: alexandra.dassonville@u-picardie.fr

The development of bacterial resistance to antibiotics (ATBs) constitutes a major threat to public health. The ESKAPEE pathogens (*E. faecium*, *S. aureus*, *K. pneumonia*, *A. baumannii*, *P. aeruginosa*, *Enterobacter* spp., *E. coli*) and *Mycobacterium tuberculosis* are a major problem in hospitals and in particular for immunocompromised patients. Moreover, very few new classes of ATBs have emerged over the last decades (Fischback, M.A., et al., *Science* **2009**, *325*, 1089–1093). Consequently, there is urgency to set up new strategies to prevent and control the emergence and spread of antimicrobial resistant micro-organisms. The range of basic mechanisms of resistance is surprisingly limited, among them diminution of ATB concentration into the bacterial cell through a reduction of cell permeability is a common way used by Gram-negative bacteria and *Mycobacterium tuberculosis*.

One promising approaches to overcome these barriers is the disruption of the bacterial membrane by natural or short synthetic antimicrobial peptides (AMPs) (Schmidt, N.W., et al., *Curr. Opin. Solid State Mater. Sci.* **2013**, *17*, 151–163). Attachment of such peptides to ATBs should improve outer membrane penetration and antibacterial efficacy ((a) Arnusch, C.J., et al., *PLoS ONE* **2012** *7*, e39768; (b) Chen, H., et al., *Mol. Pharm.* **2015**, *12*, 2505–2516; (c) Schmidt, N.W., et al., *ACS Nano* **2014**, *8*, 8786–8793).

Aminoquinolinemethanols (AQMs), synthesized in our laboratory, possess a very higher antiplasmodial activity at close to nanomolar concentrations ((a) Jonet, A., et al., EP11154229.6-2101 **2011**; (b) Jonet, A., et al., *Tetrahedron:Asymmetry* **2011**, *22*, 138–148; (c) Jonet, A., et al., *J. Antibiotics* **2013**, *66*, 683–686; (d) Bentzinger, G., et al., *Tetrahedron:Asymmetry* **2016**, *27*, 1–11). During an antibacterial screening performed in our laboratory, these AQMs showed good activities against Gram-positive bacteria (MIC = 4–16 µg/mL) and low against Gram-negative bacteria (MIC often more than 128 µg/mL). AQM-AMP conjugates with short cationic AMP sequences (4 and 6 AAs) were prepared to broaden the antibacterial spectrum of the AQM core and to delay the appearance of resistance phenomena (Yeaman, M.R., et al. *Pharmacol. Rev.* **2003**, *55*, 27–55). The synthesis and the promising antimicrobial activity of these compounds will be presented.

**Acknowledgments:** We would like to thank the Haut de France region for their financial support.

### 6.12. From Estrothiazine to Estrothiazine Sulfone: Deciphering Transcription from Cell Proliferation in Breast Cancer Cells (P16)

JacquotYves^1^*SpaggiariDanny^2^SchapplerJulie^2^LykkesfeldtAnne^3^LesniewskaEric^4^RudazSerge^2^LeclercqGuy^5^1Laboratoire des biomolécules, Université Pierre et Marie Curie—Paris 6, École normale supérieure—Paris, Centre National de la Recherche Scientifique, UMR7203 Paris, France2School of Pharmaceutical Sciences, University of Geneva & University of Lausanne, Geneva, Switzerland3Institute of Cancer Biology, Copenhagen, Denmark4Laboratoire Interdisciplinaire Carnot de Bourgogne, Université de Technologie de Belfort-Montbeliard, Université de Bourgogne, Centre National de la Recherche Scientifique, UMR-CNRS 6303 Dijon, France5Institut Jules Bordet, Brussels, Belgium*Correspondence: yves.jacquot@upmc.fr

Menopause is characterized by a panel of crippling effects that are characteristic of the decrease of the amount of the endogenous female hormone 17β-estradiol in blood. To address a better quality of life and health, hormone replacement therapy (HRT) is currently used to counteract such deleterious effects. However, HRT significantly enhances the emergence of breast tumors by ~5% in the case of estrogen-only HRT and by ~50% for combined HRT, after 10 years of treatment. This effect is principally due to the fact that drugs used in HRT activate estrogen-dependent transcription and cell proliferation. Thus, alternative strategies are required to control the carcinogenic effects associated with HRT.

Recently, we have synthesized a sulfonylated coumarin with structural analogies with coumestrol and we have tested its effects in breast carcinoma MCF-7 cells. Remarkably, this compound, which interacts directly with the estrogen receptor α (ERα) but independently from coactivators, promotes the dimerization of the receptor and activates transcription but not cell proliferation. Moreover, it preserves the turnover of the protein, revealing that this phenomenon is exclusively dependent on the ability of a molecule to induce cell proliferation. This effect, which requires ERα, depends of the presence of the sulfone function, which replaces the classical methoxy or phenolic pharmacophore of estrogens. According to in vitro binding studies and modelling calculations, this compound interacts within the binding site of 17β-estradiol, in such a way that the sulfone is engulfed within a small pocket defined by the residues Leu-346, Leu-349, Ala-350 and Leu-384.

In conclusion, our work opens new avenues for the development of estrogenic compounds that exert estrogen-dependent transcription but not cell proliferation. Such molecules could be widely used for the management of menopause and diseases that require a supplementation of estrogens. Future transcriptomic studies will be very informative to decipher the mechanism of action of this sulfone.

### 6.13. Conception of Multi-Target Directed Ligands Prodrugs to Treat Alzheimer’s Disease (P17)

ToubletFrançois-Xavier^1^LalutJulien^1^LecouteyCédric^1^HatatBérénice^1^,^2^GuieuBenjamin^1^LanthierCaroline^1^RochaisChristophe^1^DallemagnePatrick^1^1Normandie Univ, UNICAEN, Centre d’Etudes et de Recherche sur le Médicament de Normandie (CERMN), 14000 Caen, France2CNRS, UMR-5203, Institut Génomique Fonctionnelle, F-34000 Montpellier, France*Correspondence: francois-xavier.toublet@unicaen.fr

In 1906, Alois Alzheimer described the disease for the first time: the patient was suffering from particular memory disorders and present brain pathologic deposits. As a result of multiple research, the disease has proved to be more and more complex: amyloid plaque formation is due to hyperactivation of þ-secretase resulting in the formation of the amyloid peptide (Aþ). In addition, hyperphosphorylation of the tau protein was observed, causing disaggregation of microtubules, a neuronal death and a decreased cholinergic transmission (Yun, H., et al. *Exp. Neurobiol.* **2011**, *20*, 159–168).

In order to treat Alzheimer’s disease, a multitude of molecules have been synthesized but only 4 are currently marketed (Cummings, J., et al., *Res. Clin. Interv.* **2017**, *3*, 367–384): they are mostly acetylcholinesterase inhibitors (AChE), as for example rivastigmine a covalent pseudo-irreversible inhibitor, and a NMDA inhibitor, memantine.

Facing the complexity of the disease and the lack of effectiveness of the current molecules, we have developed multi-target directed ligands (MTDLs) a new strategy based on a drug with several therapeutic targets of interest to treat a disease. Thus, our laboratory synthesized donecopride (Lecoutey, C., et al., *Proc. Natl. Acad. Sci. USA* **2014**, *111*, E3825–E3830), a molecule inhibiting AChE and simultaneously activating 5-HT_4_ serotoninergic receptors.

My project consists in the development, the synthesis and the biological evaluation of new molecules with novel mechanism: prodrug MTDLs. Normally a prodrug is an inactive molecule activated by an enzyme (Rautio, J., et al. *Nat. Rev. Drug Discov.* **2008**, *7*, 255–270). In this context, the first target is inactivated by prodrug, which finds herself cut in half, releasing the second active drug.

This poster will detail the various prodrugs and explain in more details this new notion of MTDL prodrugs.


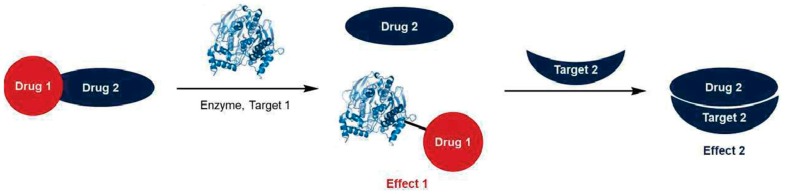


### 6.14. Structure-Guided Design of Pyridoclax Derivatives with Noxa-like Mcl-1 Binding Profile (P19)

FoghaJade^1^HedirSiham^2^,^3^De GiorgiMarcella^1^GautierFabien^4^,^5^JuinPhilippe^4^,^5^PoulainLaurent^2^,^3^Voisin-ChiretAnne Sophie^1^Sopková-de Oliveira SantosJana^1^*1Normandie Univ, UNICAEN, EA 4258 CERMN—FR CNRS INC3M, Caen, France2Normandie Univ, UNICAEN, Inserm U1086 ANTICIPE, Biology and Innovative Therapeutics for Ovarian Cancers group (BioTICLA), Centre de Lutte Contre le Cancer F. Baclesse, 3 avenue du Général Harris, 14076 Caen, France3UNICANCER, Centre de Lutte Contre le Cancer F. Baclesse, 3 avenue du Général Harris, 14076 Caen, France4Team 8 “Stress adaptation and tumor escape”, CRCINA, UMR 1232 INSERM, Université de Nantes, Université d’Angers, Institut de Recherche en Santé-Université de Nantes, Nantes, France5ICO site René Gauducheau, Boulevard Jacques Monod, Saint Herblain 44805, France*Correspondence: jana.sopkova@unicaen.fr

Protein-protein interactions are attractive targets because they control numerous cellular processes. In oncology, apoptosis regulating Bcl-2 family proteins are of particular interest. Bcl-2 proteins are crucial regulators of the intrinsic mitochondrial pathway of apoptosis and comprise both pro-apoptotic and anti-apoptotic proteins (Cory, S., et al. *Nat. Rev. Cancer* **2002**, *2*, 647–656). Apoptotic cell death is controlled via PPIs between the anti-apoptotic proteins hydrophobic groove and the pro-apoptotic proteins BH3 domain. Mcl-1 (an anti-apoptotic Bcl-2 member) is a key regulator of cancer cell survival and a known resistance factor to Bcl-2ƒBcl-x_L_ pharmacological inhibitors making it an attractive therapeutic target. As interaction among Bcl-2 family proteins occurs through a-helices, our laboratory has developed a new family of compounds able to mimic a-helix side chain distribution (abiotic foldamers) using as structural chemical units pyridine andƒor phenyl (Sopková-de Oliveira Santos et al. *J. Chem. Inf. Model*. **2012**, *52*, 429–439). The designed and synthesized oligopyridines were evaluated by their capacity to inhibit Mcl-1 in live cells and to sensitize ovarian carcinoma cells to Bcl-x_L_-targeting strategies and Pyridoclax was emerged a lead (Gloaguen, et al., *J. Med. Chem.*, **2015**, *58*, 1644–1668; Poulain et al. M Patent EP14305309.8. **2014)**. Here, using a structure-guided design from the Pyridoclax, we identified a novel selective Noxa-like Mcl-1 inhibitor, MR31367 that selectively binds to Mcl-1 hydrophobic groove and releases Bak and Bim from Mcl-1.

### 6.15. Alzheimer’s Disease: Development of a Multistep Procedure to Characterize Modulators of Amyloid Peptide Aggregation (P20)

SmeraldaWilly^1^*SinceMarc^1^BoisserieMichael^2^CorvaisierSophie^1^CardinJulien^2^Malzert-FréonAurélie^1^1Centre d’Etudes et de Recherche sur le Médicament de Normandie (CERMN), Normandie Univ, UNICAEN, 14000 Caen, France2NIMPH Team, CIMAP CNRS UMR6252, EnsiCaen-UNICAEN-CEA, 14050 Caen Cedex, France*Correspondence: willy.smeralda@unicaen.fr

Alzheimer’s disease (AD) is the most widespread form of senile dementia worldwide and represents the leading socioeconomic problem in healthcare. The onset and the progression of this neurodegenerative disease is associated with the aggregation of the amyloid-þ peptide (Aþ) through a fibrillization process. Hence, an attractive therapeutic strategy against this disorder is the development of molecules able to interfere at specific steps of this fibrillization pathway (Habchi, J., et al. *Proc. Natl. Acad. Sci. USA* **2017**, *114*, E200–E208), i.e., able to inhibit the formation of the soluble toxic oligomeric Aþ aggregates. To identify such promising therapeutic molecules, experimental methods are required to precisely follow and characterize the state (monomer ƒ oligomers ƒfibrils) and the conformation of Aþ peptide during its fibrillization process. In the same time, these methods must be simple enough to remain compatible with the High Throughput Screening (HTS) of new compounds with potential interest in AD treatment.

In the present study, we propose to combine experimental methods to permit a multiparameter characterization of potential modulators of Aþ_1-42_ fibrillization. These kinetic studies are carried out in presence of liposomes as biomimetic neuronal membranes. Indeed, it has been established that lipids of the neuronal membrane are an important factor in the cascade of events that leads to the formation of the amyloid fibers.

Thus, fluorescence of Thioflavin T, a specific dye for amyloid species (Biancalana, M., et al., *Biochim. Biophys. Acta BBA—Proteins Proteomics* **2010**, *1804*, 1405–1412), was kinetically monitored to follow the Aþ fibrillization pathway. In parallel, considering that the toxic fibers formed by Aþ are mainly organized into þ-sheets, assays were correlated with the secondary structure analysis of the peptide using ATR-FTIR spectroscopy (Sarroukh, R., et al. *Biochim. Biophys. Acta BBA—Biomembr.* **2013**, *1828*, 2328–2338). Moreover, a liposome leakage assay supported by dynamic light scattering (DLS) studies was carried out to evaluate the impact of the interactions between the peptide and membranes to predict any destabilization effect.

From results obtained to date, it appears that the tested modulators present a range of effects that can affect defined stages of the aggregation of the Aþ_1-42_ peptide, organized in defined secondary structure. This study highlights also some advantages of the use of liposomes to characterize and discriminate newly Aþ aggregation modulators synthetized by chemists and could even be expanded to other amyloid diseases.

### 6.16. Synthesis of Guanylated Hydroxyethylene Peptide Isosteres for Inhibition of FXIIa (P21)

SimonFrançoisPierardSteffyPochetLionelLannersSteve*Namur Medicine & Drug Innovation Center (NAMEDIC), Namur Research Institute in Life Science (NARILIS), University of Namur, B-5000 Namur, Belgium*Correspondence: steve.lanners@unamur.be

A great challenge in anticoagulation therapy is to limit thrombosis without causing increased risk of bleeding in patients. Oral anticoagulants have come closer to this goal but hemorrhage is still reported. Coagulation factors are further investigated as potential targets to inhibit. Among them, FXIIa, a serine protease, appears to be promising according to numerous animal studies (Weitz, I., et al., *J. Front. Med.* **2017**, *4*, 19). Up to now, FXIIa inhibitors under development include proteins, peptides and RNA-based macromolecules. (Larsson, M., et al., *Sci. Transl. Med.* **2014**, *6*, 222ra17). On the contrary, low-molecular-weight inhibitors are overlooked. Our goal is to synthesize and assess small molecules as potential FXIIa inhibitors. Based on a 3D model of FXIIa developed in our laboratory, we decided to investigate two series of molecules, including chiral compounds **1**.


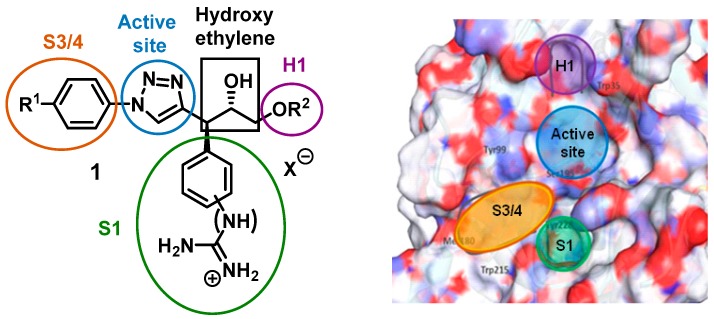


Each fragment was selected to fit one of the binding pockets around the active site of FXIIa. The chiral hydroxyethylene group was added to mimic the tetrahedral intermediate involved in serine protease enzymes. In this work, the synthesis of model compounds is discussed.

**Acknowledgments:** This work was supported by a research fellow grant from the F.R.S.-FNRS.

### 6.17. Computational ‘Microscopy’ of Pyridoclax—Mcl-1 Interaction (P22)

BenabderrahmaneMohammed*DenisCamilleVoisin-ChiretAnne-SophieBureauRonanSopkova-de Oliveira SantosJanaCentre d’Etudes et de Recherche sur le Médicament de Normandie (CERMN), Normandie Univ, UNICAEN, 14000 Caen, France*Correspondence: mohammed.benabderrahmane@unicaen.fr

Myeloid cell leukemia-1 (Mcl-1) is an anti-apoptotic member of the BCL-2 family of proteins. It is often over-expressed in human cancers. Pyridoclax (Poulain, L., et al. EP14305309, 04/03/201) is an original oligopyridine lead, very promising in treatment of chemoresistant cancers. Using different biological and biophysical tests (Gloaguen, C., et al., *J. Med. Chem.* **2015**, *58*, 1644–1668), it was identified as a selective Mcl-1 inhibitor.

Computational ‘microscopy’ refers to the use of computational resources to simulate the dynamics of a molecular system. This computational ‘microscopy’ technique is able to capture the interplay between a protein and a ligand at a spatio-temporal resolution that is unmatched by other methods. In this work, the mechanism of the interaction between pyridoclax and Mcl-1 key-residues is unraveled using a combined molecular docking/dynamics investigation.


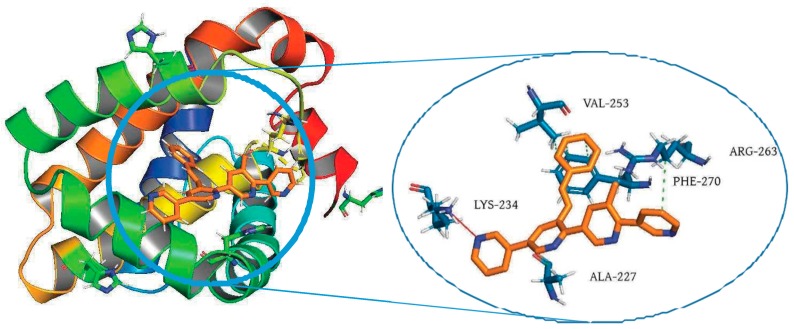


(Left) Pyridoclax (light brown & licorice representation) interacting within the binding groove of Mcl-1 (cartoon representation). (Right): detailed view of Mcl-1 interacting residues: H-bond (solid red) & hyrdrophobic interactions (dashed green) represent the main contribution.

### 6.18. Synthesis and Biological Evaluation of Functionalized 4,5-dihydropyridazinones as Anti-Inflammatory Agents (P23)

Allart-SimonIngrid^1^MoniotAurélie^2^BarberotChantal^1^MalleretLaurette^3^GuillaumeChristine^2^BentaherAzzaq^3^GangloffSophie^2^HénonEric^1^VelardFrédéric^2^SapiJanos^1^GérardStéphane^1^*1Université de Reims Champagne-Ardenne, Institut de Chimie Moléculaire de Reims (ICMR), UMR CNRS 7312, UFR Sciences, Moulin de la housse and UFR Pharmacie, 51 rue cognacq-jay, 51096 Reims, France2Université de Reims-Champagne-Ardenne, EA 4691 Biomatériaux & Inflammation en site OSseux (BIOS), UFR Pharmacie, 51 rue cognacq-jay, 51096 Reims, France3Centre International de Recherche en Infectiologie (CIRI), EA7426, Faculté de Médecine Lyon-Sud, 165 chemin du Grand Revoyet, 69921 Oullins, France*Correspondence: stephane.gerard@univ-reims.fr

In the recent years, cyclic nucleotide phosphodiesterase type 4 (PDE4), which controls intracellular level of cyclic nucleotide cAMP, has emerged as a suitable target for anti-inflammatory therapy (Mulhall, A.M., et al., *Expert Opin. Invest. Drugs* **2015**, *24*, 1597–1611; Michalski, J.M., et al., *Clin. Pharmacol. Ther.* **2012**, *91*, 134–142).

In continuation of our efforts to develop PDE4 inhibitors for the treatment of respiratory diseases (Gérard, S. et al. Pat. Appl. WO 2016066973 A1 20160506), we describe the design and the synthesis of a new family of compounds possessing pyridazinone scaffold, corresponding molecular modeling study and their evaluation as anti-inflammatory agents. Among these compounds, two 4,5-dihydropyridazinone derivatives possess promising activity, selectivity towards PDE4 isoenzyme and are able to reduce IL-8 production (Barberot, C., et al., *Eur. J. Med. Chem.* **2018**, *146*, 139–146).


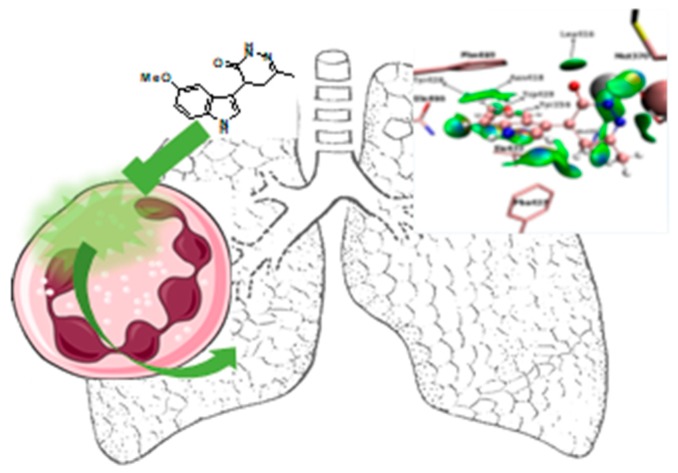


**Acknowledgments:** Financial support from the French National Centre for Scientific Research (CNRS), the Regional Council of Champagne–Ardenne (France), the French Ministry of Higher Education and Research (MESR) and the European Regional Development Fund (FEDER) to the PlAneT CPER project is acknowledged. C.B. thanks the Regional Council of Champagne–Ardenne (France) for a Ph.D. fellowship. A.M. Ph.D. fellowship is co-funded by Reims-Metropole and the European Union (*Europe invests in Champagne-Ardenne with the European Regional Development Fund).* Thanks are given to the Maison de la simulation de Champagne-Ardenne (Reims, France) for the provision of computational facilities.

### 6.19. Structural and Inhibition Study of Mycobacterium Tuberculosis Phosphoserine Phosphatase (SerB) (P25)

HaufroidMarie*PiersonEliseCallaertsNephtaliMirgauxManonWoutersJohanLaboratoire de Chimie Biologique Structurale (CBS), Université de Namur (UNamur) and Namur Medicine & Drug Innovation Center (NAMEDIC-NARILIS)*Correspondence: marie.haufroid@unamur.be

Tuberculosis is still the leading cause of death by a single treatable infectious disease, since it kills over 1.5 million people every year. Drug resistant and multi-drug resistant strains are emerging and treatment options are limited. The search for new targets and drugs is needed (Global Tuberculosis Report 2017. *World Health Organization Online*, www.who.int).

In the search for new targets essential for bacterial survival, it was shown that SerB2 (a phosphoserine phosphatase) is secreted within cytoplasm of THP-1 macrophages infected with tuberculosis. The enzyme can dephosphorylate proteins and transcription factors in order to cause cytoskeleton rearrangement and deactivate the host immune response (Sharma, A.K., et al., *Indian J. Microbiol.* **2016**, 1–10). The inhibition of MtSerB2 may help to improve treatment options against the disease.

SerB2 is part of the serine pathway, which produces the amino-acid L-serine from D-3- phosphoglycerate, an intermediate of glycolysis. It catalyses the third and last step of this pathway which is the transformation of phospho-L-serine into L-serine. There are only a few described inhibitors of MtSerB2 and the crystallographic structure of the protein is still unknown (Arora, G., et al., *J. Biol. Chem*. **2014**, *289*, 25149–25165).

The purpose of this work is to study the structure of the protein and to conceive new inhibitors. In order to do that, an enzyme inhibition assay was optimized and performed to allow a screening of NAMEDIC’s chemical library. On 125 tested compounds, three showed good inhibitions profile and were found to be competitive inhibitors by kinetic studies. Crystallization assays and docking experiments are still ongoing in order to elucidate the pose of those inhibitors within the active site. Furthermore, in order to assess and design selective inhibitors, human phosphoserine phosphatase (hPSP) was also produced and inhibition and crystallization assays were also performed.

**Acknowledgments:** The authors thanks members of NAMEDIC who generated NAMEDIC library, in particular Prof B. Masereel and L. Pochet.

### 6.20. Bioproduction of Polymyxin Derivatives (P26)

SalsanoCaroline^1^TambadouFatoumata^1^SacariasJ.E. Pereanez^2^BarthélémyCyrille^1^DidelotSandrine^1^SopénaValérie^1^ThiéryValérie^1^*ChevrotRomain^1^1University of La Rochelle, UMR CNRS 7266, La Rochelle, France2Autonomous University of Campeche, Department of Environmental Microbiology and Biotechnology, Mexico*Correspondence vthiery@univ-lr.fr

With the rise in bacterial resistance and overuse of antibiotics, it is necessary to find efficient treatments against human infections. One strategy is to enhance existing molecules by genetic engineering. As part of our ongoing research (Chevrot, R., et al., *Probiotics Antimicrob. Proteins* **2013**, *5*, 18–25; Tambadou, F., et al., *Arch. Microbiol*, **2015**, *197*, 521–532), we respectively isolated from the environment and a sputum of a cystic fibrosis patient two novel antibiotic producing strains of *Paenibacillus*, B-LR and P-32. *Paenibacilli* are ubiquitous bacteria that produce antibiotics such as polymyxins and fusaricidins. These molecules are obtained from specific enzymes called Nonribosomal Peptide Synthetases (NRPS). The strains B-LR and P-32 inhibit the growth of *Pseudomonas aeruginosa* and *Staphylococcus aureus* clinical strains (including methicillin resistant strains). These peptides were purified and characterized by LC-MS. B-LR produces polymyxin E (colistin) and ten different molecules while P-32 synthesizes a new depsipeptide. Two genomic libraries were constructed and screened in order to find the gene clusters responsible for the biosynthesis of these molecules. This screening lead to the discovery of the colistin gene cluster in B-LR. This new gene cluster covers 41 kb and includes 5 ORF. Three of them encode for NRPS involved in the colistin synthesis, whereas two others are ABC transporter-like genes that may release the antibiotic. This study constitutes the first description of the biosynthesis pathway of this commercial antibiotic that could contribute to a better understanding of the biosynthesis. The biotechnological development appears to be a good way the pharmaceutical production of optimized antibacterial treatments.

### 6.21. Discovery of a Novel Series of Inhibitors of Anti-Apoptotic Proteins for Treatment of Chemorestistant Ovarian Cancers (P29)

DenisCamille^1^*De PascaleMartina^1^MarekhaBogdan^1^JouanneMarie^1^KiefferCharline^1^BrotinEmilie^2^WeiswaldLouis-Bastien^2^DenoyelleChristophe^2^PoulainLaurent^2^AziezAsma Bourafai^3^SebbanMuriel^3^OulyadiHassan^3^BureauRonan^1^ChiretAnne Sophie Voisin^1^1Centre d’Etudes et de Recherche sur le Médicament de Normandie, Normandie Univ, UNICAEN, CERMN, 14000 Caen, France2Normandie Univ, UNICAEN, INSERMN U10186 « ANTICIPE » Axe 2 Bioticla, Centre François Baclesse, Caen, France3Normandie Univ, UNIROUEN, INSA Rouen, CNRS, COBRA UMR 6014, F-76000 Rouen, France*Correspondence: camille.denis@unicaen.fr

Protein-protein interactions (PPIs) control many important physiological processes within human cells. A hallmark of cancers is the evasion of apoptosis, which is often associated with the upregulation of the anti-apoptotic members of the Bcl-2 family of proteins. These proteins comprise pro-survival (Bcl-2, Bcl-x_L_, Mcl-1) and pro-apoptotic members. In many cancers, the balance between the pro- and anti-apoptotic Bcl-2 family members is tipped towards survival. Drugs inhibiting the pro-survival activity of Bcl-2 proteins to restore cell death may therefore be valuable as cancer therapeutics.

Even though potent and selective inhibitors of Bcl-2 and Bcl-x_L_ have been developed, it is not enough to re-establish the apoptosis. Mcl-1 protein has been shown to cause resistance to chemotherapeutics. Consequently, the inhibition of this protein is required to maintain activity. Our laboratory has synthesized a selective inhibitor of Mcl-1 protein, pyridoclax (Gloaguen, C., et al., *J. Med. Chem.* **2015**, *58*, 1644–1668). However, the discovery of dual Mcl-1 and Bcl-x_L_ inhibitors would be an important advance in cancer treatment.

Starting from the structure of our lead compound, pyridoclax, fragments have been designed with the objective to retain an activity against Mcl-1 and concomitantly target Bcl-x_L_. The design, synthesis and the first biological results of these compounds will be presented in this poster.

### 6.22. β-Lactam Analogues of Combretastatin A-4 Prevent Metabolic Inactivation by Glucuronidation in Chemoresistant HT-29 Colon Cancer Cells (P30)

MalebariAzizah^1^,^2^*NathwaniSeema M.^3^FayneDarren^4^O’BoyleNiamh M.^2^MeeganMary J.^2^1Department of Pharmaceutical Chemistry, College of Pharmacy, King Abdulaziz University, Jeddah, KSA2School of Pharmacy and Pharmaceutical Sciences, Trinity Biomedical Sciences Institute, Trinity College Dublin, Ireland3School of Biochemistry and Immunology, Trinity Biomedical Sciences Institute, Trinity College Dublin, Ireland4Molecular Design Group, School of Biochemistry and Immunology, Trinity Biomedical Sciences Institute, Trinity College Dublin, Ireland*Correspondence: melibaa@tcd.ie

Glucuronidation by uridine 5-diphosphoglucuronosyl transferase enzymes (UGTs) is a cause of intrinsic drug resistance in cancer cells (Aprile, S., et al., *Drug Meta. Dispos.* **2007**, *35*, 2252–2261). Glucuronidation of combretastatin A-4 (CA-4) was previously identified as a mechanism of resistance in hepatocellular cancer cells (Quan, H., et al., *J. Pharmacol. Exp. Ther.* **2009**, *330*, 326–333). Herein, we propose chemical manipulation of þ-lactam bridged analogues of Combretastatin A-4 as a novel means of overcoming drug resistance associated with glucuronidation due to the expression of UGTs in the CA-4 resistant human colon cancer HT-29 cells. The alkene bridge of CA-4 is replaced with a þ-lactam ring to circumvent potential isomerisation while the potential sites of glucuronate conjugation are deleted in the novel 3- substituted- 1,4-diaryl-2-azetidinone analogues of CA-4. We hypothesise that glucuronidation of CA-4 is the mechanism of drug resistance in HT-29 cells. Ring B thioether containing 2-azetidinone analogues of CA-4 such as 4-(4-(methylthio)-phenyl)-3-phenyl-1-(3,4,5-trimethoxyphenyl)azetidin-2-one was identified as the most potent inhibitors of tumour cell growth, independent of UGT status, displaying antiproliferative activity in the low nanomolar range. These compounds also disrupted the microtubular structure in MCF-7 and HT-29 cells, and caused G2ƒM arrest and apoptosis. Taken together, these findings highlight the potential of chemical manipulation as a means of overcoming glucuronidation attributed drug resistance in CA-4 resistant human colon cancer HT-29 cells, allowing the development of therapeutically superior analogues.


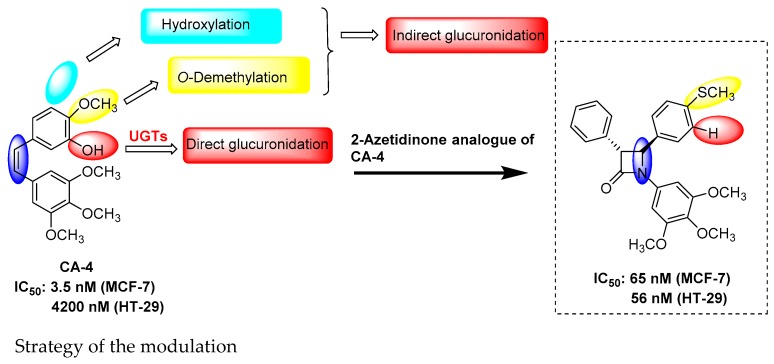


### 6.23. The Phosphorylation of the Residues Ser-202, Thr-205 and Ser-208 of the Protein Tau Is the Driving Force for Its Aggregation, an Observation of Clinical Interest in the Context of Alzheimer Disease (P33)

DespresClément^1^ByrneCillian^2^,^3^QiHaoling^1^CantrelleFrançois-Xavier^1^HuventIsabelle^1^ChambraudBéatrice^2^,^4^BaulieuEtienne-Emile^2^,^4^*JacquotYves^3^*LandrieuIsabelle^1^LippensGuy^5^*Smet-NoccaCaroline^1^*1Unité de Glycobiologie Structurale et Fonctionnelle, Centre National de la Recherche Scientifique (CNRS), UMR8576, Université de Lille, Institut National de la Recherche Agronomique (INRA), USC1409, Lille, France2Institut Baulieu, Université Paris-Saclay, Institut National de la Santé et de la Recherche Médicale (INSERM), UMR1195, Kremlin-Bicêtre, France3Laboratoire des biomolécules, Université Pierre et Marie Curie—Paris 6, École normale supérieure (ENS), Centre National de la Recherche Scientifique (CNRS), UMR7203 Paris, France4Petites molécules de neuroprotection, neurodégénération, neurogénération et remyélinisation, Institut National de la Santé et de la Recherche Médicale (INSERM), UMR1195, Le Kremlin-Bicêtre, France5Laboratoire d’ingénierie des Systèmes Biologiques et des Procédés - Institut National de la Recherche Agronomique (INRA), UMR0792, Institut National des Sciences Appliquées—Centre National de la Recherche Scientifique (CNRS), UMR5504, Toulouse, France*Correspondence: glippens@insa-toulouse.fr; etienne.baulieu@inserm.fr; yves.jacquot@sorbonne-universite.fr; caroline.smet@univ-lille1.fr

Alzheimer’s disease is characterized by two important cytological hallmarks: (i) extraneuronal Aβ amyloid fibers and (ii) intraneuronal aggregates (tangles) that are principally composed of the hyperphosphorylated (pathological) form of Tau. In a physiological context, the protein Tau is in charge of the stabilization of microtubules and is thus considered as a microtubule-associated protein (MAP). Briefly, Tau is composed of 441 amino acids amongst which are 85 real or potential phosphorylation sites (85 serines / threonines). Phosphorylation sites seem to be of prime importance pathologically. For example, the doubly phosphorylations at the Ser-202 and Thr-205, which induce a turn-like structure, are components of the epitope that is recognized by the AT8 antibody, which is used to diagnose Alzheimer disease post-mortem (Gandhi, N.S., et al. *Angew. Chem. Int. Ed.* **2015**, *54*, 6819–6823).

We show here not only that hyperphosphorylation is a switch for Tau aggregation, but also that the phosphorylation at the three positions Ser-202, Thr-205 and Ser-208, with absence of phosphorylation at the Ser-262, is sufficient to induce Tau aggregation, in vitro.


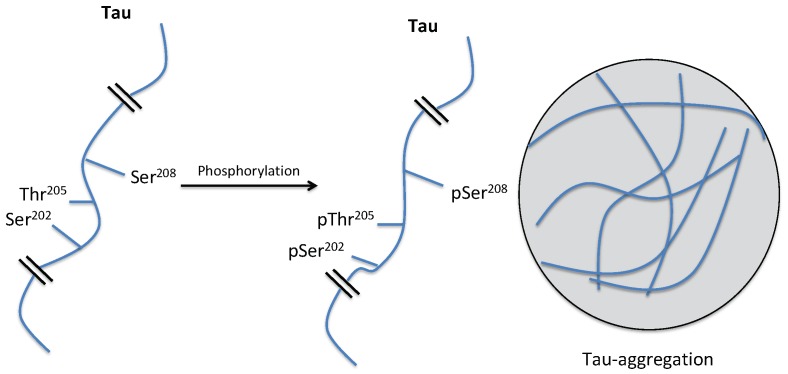


Whereas the Thr-205 is phosphorylated by the serine/threonine kinase ERK2, the Ser-208 is phosphorylated by others kinases. Thus, targeting specifically the intraneuronal kinases that are in charge of the phosphorylation of these three residues could be of therapeutic interest (Despres, C., et al. *Proc. Natl. Acad. Sci. USA* **2017**, *114*, 9080–9085).

**Acknowledgments:** This work was supported by grants from LabEx DISTALZ, the Région Hauts-de-France, the Centre National de la Recherche Scientifique, the TGE RMN THC, the Fédération de Recherche FRABio, MetaToul (MetabolHUB-AR-11-INBS-0010) and the Institut Baulieu.

### 6.24. Modulation of Solubility of Trisubstituted Harmine Derivatives Using Crystallization Engineering (P33)

MarxSébastien*BodartLaurieMeinguetCélineTumanovNikolayWoutersJohanNamur Medicine & Drug Innovation Center (NAMEDIC-NARILIS) Département de Chimie, Laboratoire de Chimie Biologique Structurale (CBS), Université de Namur, 5000 Namur, Belgium*Correspondence: sebastien.marx@unamur.be

Harmine is a natural β-carboline compound which can be isolated from *Peganum harmala*, presenting antiproliferative activity. Trisubstituted harmine derivatives were shown to be more potent than mono- and di-substituted ones (Meinguet, C., et al., *Eur. J. Med. Chem*., **2015**, *94*, 45–55; Meinguet, C., et al., *Eur. J. Pharm. Sci.*, **2015**, *77*, 135–140). Here, new trisubstituted harmine derivatives were designed and synthetized in our group with the aim to overcome the intrinsic resistance of cancer cells to apoptotic stimuli.

However, trisubstituted harmine derivatives often present a moderate solubility at pH 7.4. In this context cyclodextrin complexes with CM16 lead compounds were already prepared in our group to improve compounds solubility (Meinguet, C., et al. *Eur. J. Pharm. Sci.* **2015**, 77, 135–140). Here we propose a new approach to reach this objective. Indeed, the third substituent chosen to be placed on the harmine core contains a pyridine moiety in order to improve compound solubility in comparison with benzyl moiety. Moreover, pyridine increases the tendency towards cocrystal/salt formation which is also considered as a potential method to modulate the solubility of the new analogs of CM16 (*Pharmaceutical Salts and Co-crystals*, J. Wouters and L. Quéré, eds. RSC Publishing: city, country, 2012, pp. 116–119). The aim of this work is to obtain novel druggable harmine-based molecules combining antiproliferative activity in micromolar to submicromolar range and a high solubility at physiological pH in order to enable intravenous injection of these compounds.

**Acknowledgments:** SM and LB thank the Fonds National pour la Recherche Scientifique-FNRS for financial support and the PC2 technological platform of UNamur.

**Funding:** The authors would like to acknowledge the Labex SynOrg, Sponsors & Exhibitors for financial support. For more details, please visit: http://gp2a.org/.

## 7. Conclusions

The meeting was a real success with more than 110 attendees that had the opportunity of scientific exchanges through 14 lectures, five young researcher communications, 19 flash PhD communications and 53 posters.

By sharing experiences and expertise in wide range of topics related to medicinal chemistry, new collaborations were discussed to start in the near future. Dr. Jade Fogha (University of Orléans, France) received the award for the best young researcher communication, Reynald Mangeant (University of Caen, France) for the best poster with flash oral presentation (sponsored by *Pharmaceuticals*, a journal published by MDPI).

Florence Couly (University of Rouen, France) and Birgit Gaiser (University of Copenhagen, Denmark) received awards for best posters (Sponsored by ManRos Therapeutics, Roscoff, France).

The 27th Annual GP2A Medicinal Chemistry Conference is scheduled for 21st–23rd August 2019 in Nottingham, UK.

The GP2A committee and the local organizing committee thank Universities of Caen and Rouen, and all the sponsors and exhibitors for supporting this annual event.

